# Extended spectrum beta lactamases and carbapenemase-producing *Escherichia coli* in humans, animals and the environment in East Africa: a scoping review

**DOI:** 10.1016/j.onehlt.2026.101606

**Published:** 2026-10-10

**Authors:** Magreth Erick Macha, Alisen Ayitewala, Damalie Nakanjako

**Affiliations:** a St. Francis University College of Health and Allied Sciences, Ifakara, Tanzania; b African Research Universities Alliance (ARUA), Cluster of Research Excellence (CoRE) in Advanced Infectious Diseases Research and Training, Makerere University College of Health Sciences, Kampala, Uganda; c Central Public Health Laboratories (CPHL), National Health Laboratory and Diagnostic Services (NHLDS), Ministry of Health, Kampala, Uganda; d Department of Microbiology, School of Biomedical Science, Makerere University College of Health Sciences, Kampala, Uganda; e Department of Medicine, School of Medicine, Makerere University College of Health Sciences, Kampala, Uganda; f Infectious Diseases Institute, Makerere University College of Health Sciences, Kampala, Uganda; g Africa-Europe Cluster of Excellence in Translational Science in Infection, Immunity and Inflammation (CoRE Triii), Makerere University College of Health Sciences, Kampala, Uganda

**Keywords:** *Escherichia coli*, ESBL, Carbapenemase, Genomics, Surveillance, Human, Animal, Environment, One health, East Africa

## Abstract

**Background:**

Antimicrobial resistance (AMR) is escalating in East Africa through close human-animal-environment interactions. Extended-spectrum beta-lactamases (ESBL) and carbapenemase-producing *Escherichia coli* are clinically critical pathogens, yet local evidence is limited. This scoping review synthesizes evidence on ESBL and carbapenemase-producing *E. coli* in East Africa to inform One Health AMR control strategies.

**Methods:**

Following PRISMA-ScR guidelines, PubMed and Scopus were searched, identifying 279 records published between 2015 and 2025. After removing duplicates and screening, 185 articles were assessed for eligibility; 154 studies spanning human, animal and environmental sources met the inclusion criteria. Two reviewers independently extracted epidemiological, microbiological and molecular data in Covidence, including resistance genes and transmission evidence.

**Results:**

The 154 included studies spanned 11 East African countries, led by Ethiopia (*n* = 55), Tanzania (*n* = 39) and Uganda (*n* = 22). Most studies (85.7%) were cross-sectional and human-focused (68.2%), with only 19 (12.3%) using a multi-sector One Health design. Overall, 21,554 *E. coli* isolates were recovered from 74,700 samples. ESBL prevalence was reported in 117 studies (76.0%, median 46.8%) and carbapenemase prevalence in 65 (42.2%, median 3.3%), representing heterogeneous study-level estimates. *bla*_CTX-M_ predominated (89.9%), led by *bla*_CTX-M-15_ (26.1%); reported carbapenemase genes included *bla*_NDM_, *bla*_OXA-48_, *bla*_OXA-181_ and *bla*_KPC._ Whole-genome sequencing in 30 studies (19.5%) identified high-risk clones ST131, ST10, ST38 and ST410, though animal, food-chain and environmental sectors remained underrepresented.

**Conclusion:**

This review maps the current evidence on landscape for ESBL/carbapenemase- producing *E. coli* in East Africa, highlighting gaps in surveillance that are largely due to heterogeneity of available genomics data and limited longitudinal data within the “One Health” sectors. We recommend scaling up genomic capacity and standardized longitudinal surveillance to better understand national and regional AMR transmission and MDR *E. coli* infections.

## Highlights

• ESBL-producing *E. coli* is widespread across East African One Health sectors.
• *bla*_CTX-M-15_ and IncF plasmids were the most frequently reported.
• High-risk clones ST131, ST10, ST38 and ST410 exist across human, animal and environmental sectors.
• Limited genomic surveillance constrains evidence for cross-sector transmission.

## 1 Introduction

Antimicrobial resistance (AMR) is a major global health threat, with sub-Saharan Africa bearing the highest burden despite limited surveillance capacity [1], [2]. Without effective interventions, AMR is projected to cause 1.91 million deaths annually by 2050 and 39.1 million cumulative deaths between 2025 and 2050 [3]. *Escherichia coli* is a common commensal bacterium of the human and animal gastrointestinal tract, but it is also an important opportunistic pathogen causing diarrhoeal disease and extra-intestinal infections, particularly urinary tract and bloodstream infections [4], [5]. It is the leading bacterial pathogen associated with AMR attributable deaths globally, with resistance to third-generation cephalosporins increasing across all world health organizations (WHO) regions, including Africa [3], [6]. Although African data remain limited, available evidence suggests resistant infections are associated with greater morbidity and mortality than susceptible infections [7], [8]. Extended-spectrum β-lactamase (ESBL) and carbapenemase-producing *E. coli* are a major public health concern because they confer resistance to third-generation cephalosporins and carbapenems, which are important treatment options for severe infections in low and middle-income countries. Reflecting this threat, WHO has classified ESBL-producing *E. coli* as a critical-priority pathogen [9]. Their global spread is driven by high-risk clones, particularly ST131 and horizontal transfer of resistance genes such as *bla*_CTX-M-15_ [10], [11].

In East Africa, close and frequent contact between humans, animals and the environment creates conditions that facilitate the emergence and transmission of these resistant strains, while reports of carbapenemase-producing *E. coli* are increasing [12], [13]. Discerning the full diversity of ESBL and carbapenemase-producing *E. coli* across ecological niches and geographic settings is essential for identifying reservoirs and transmission pathways. However, evidence from East Africa remains fragmented, with a strong focus on human clinical isolates, limited data from animal, food-chain and environmental sources and scarce genomic evidence of cross-sectoral transmission [14], [15]. This scoping review addresses this gap by synthesizing evidence on ESBL and carbapenemase-producing *E. coli* across human, animal and environmental sectors in East Africa over the past decade. It characterizes regional prevalence, dominant resistance mechanisms and high-risk clones and maps the current state of One Health transmission evidence, associated risk factors including sequence types (STs) and key knowledge gaps, to inform regional surveillance and containment strategies.

## 2 Methodology

### 2.1 Study design

This scoping review followed the Preferred Reporting Items for Systematic Reviews and Meta-Analyses extension for Scoping Reviews (PRISMA-ScR) [16]. The research question, objectives, inclusion and exclusion criteria were discussed and defined by all authors.

### 2.2 Search strategy

PubMed and Scopus were searched for studies published between January 2015 and December 2025, combining keywords, Medical Subject Headings (MeSH) and Boolean operators (AND/OR) related to ESBL, carbapenemase, *E. coli,* antimicrobial resistance, One Health and East African countries. East African countries (defined per African Union regional classification and geographic location [17]: Burundi, Comoros, Djibouti, Eritrea, Ethiopia, Kenya, Madagascar, Mauritius, Rwanda, Seychelles, Somalia, Sudan, South Sudan, Tanzania and Uganda). Full search strings are in **Table S1**.

PubMed and Scopus were selected for their broad coverage of biomedical and microbiological literature and to enable feasible dual-reviewer screening within our available resources.

### 2.3 Study selection and eligibility criteria

Records were deduplicated in Mendeley and screened in Covidence [18] by two independent reviewers (title and abstract, followed full-text review), with disagreements resolved through discussion with a third reviewer. Of 279 records identified, 185 underwent full-text review and 154 studies met inclusion criteria (Fig. 1). Eligible studies reported ESBL and carbapenemase-producing *Escherichia coli* from human, animal and/or environmental samples across East African countries, using phenotypic and/or molecular detection methods, with extractable data, published in English (2015–2025); reviews, editorials, conference abstracts and studies lacking *E. coli*-specific data were excluded.

**Fig. 1:**
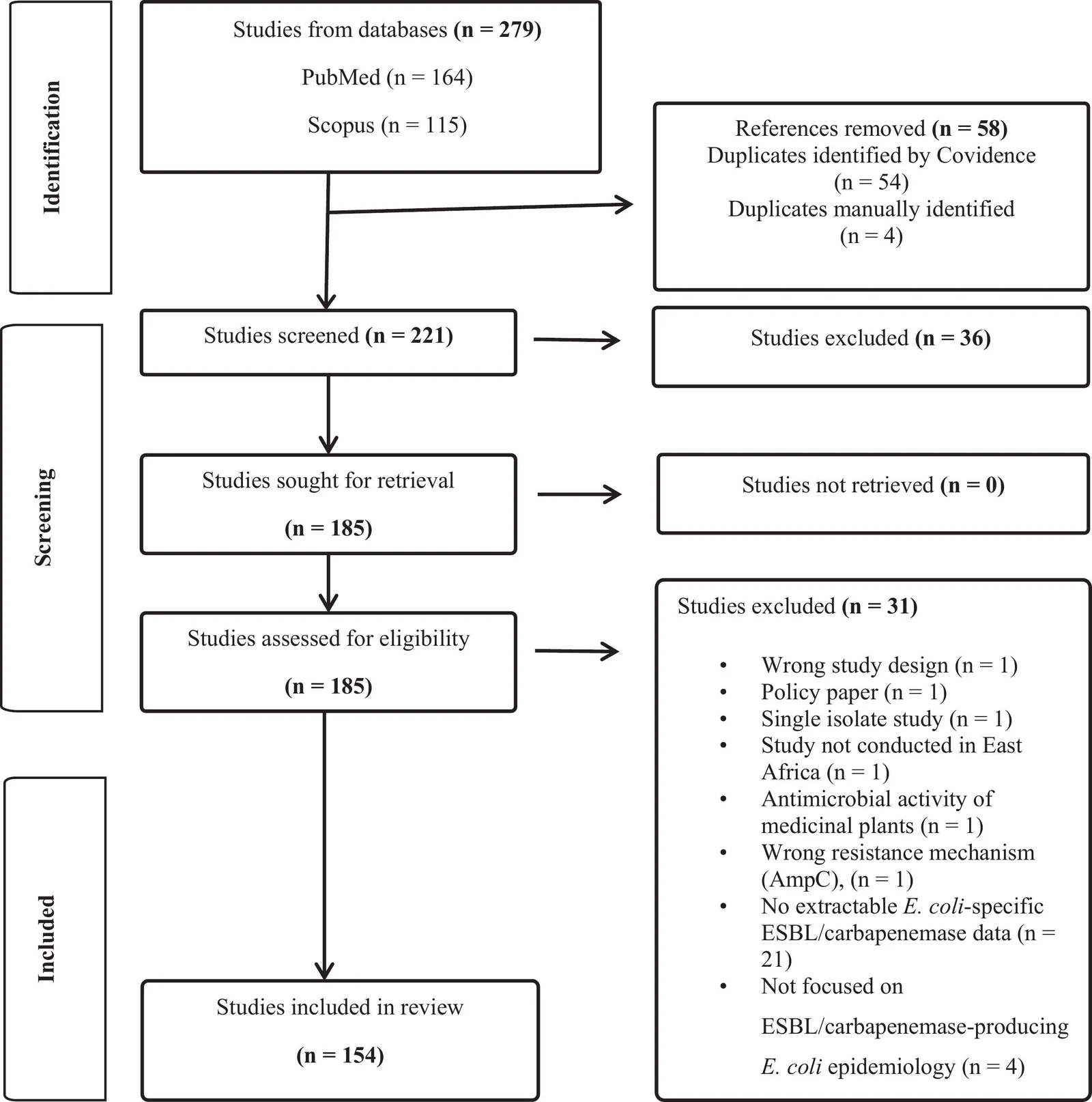
PRISMA-ScR flow diagram of study identification, screening and inclusion, resulting in 154 included studies.

### 2.4 Data extraction

A standardized data extraction form captured: study identification, design and setting, population, sample and isolate characteristics, diagnostic and susceptibility-testing methods, molecular characteristics, resistance prevalence, risk factors, transmission pathways and study limitations (full variable list, **Table S2**).

### 2.5 Data analysis

Extracted data were exported from Covidence, cleaned and coded in Microsoft Excel, and summarized using descriptive statistics and narrative synthesis, presented as tables, figures, maps and text. Consistent with the Joanna Briggs Institute methodology and the PRISMA Extension for Scoping Reviews (PRISMA-ScR), formal critical appraisal of the included studies was not undertaken, as the aim was to map available evidence rather than assess methodological quality.

## 3 Results

### 3.1 Geographic distribution of included studies

The 154 studies were unevenly distributed across 11 East African countries (Fig. 2). Ethiopia contributed 55 (36%), Tanzania 39 (25%) and Uganda 22 (14%), together accounting for 75% of studies. Kenya, Rwanda, Madagascar, Sudan, Djibouti, Somalia, Burundi and Eritrea contributed fewer. No eligible studies were identified from Comoros, Mauritius, Seychelles or South Sudan.

**Fig. 2:**
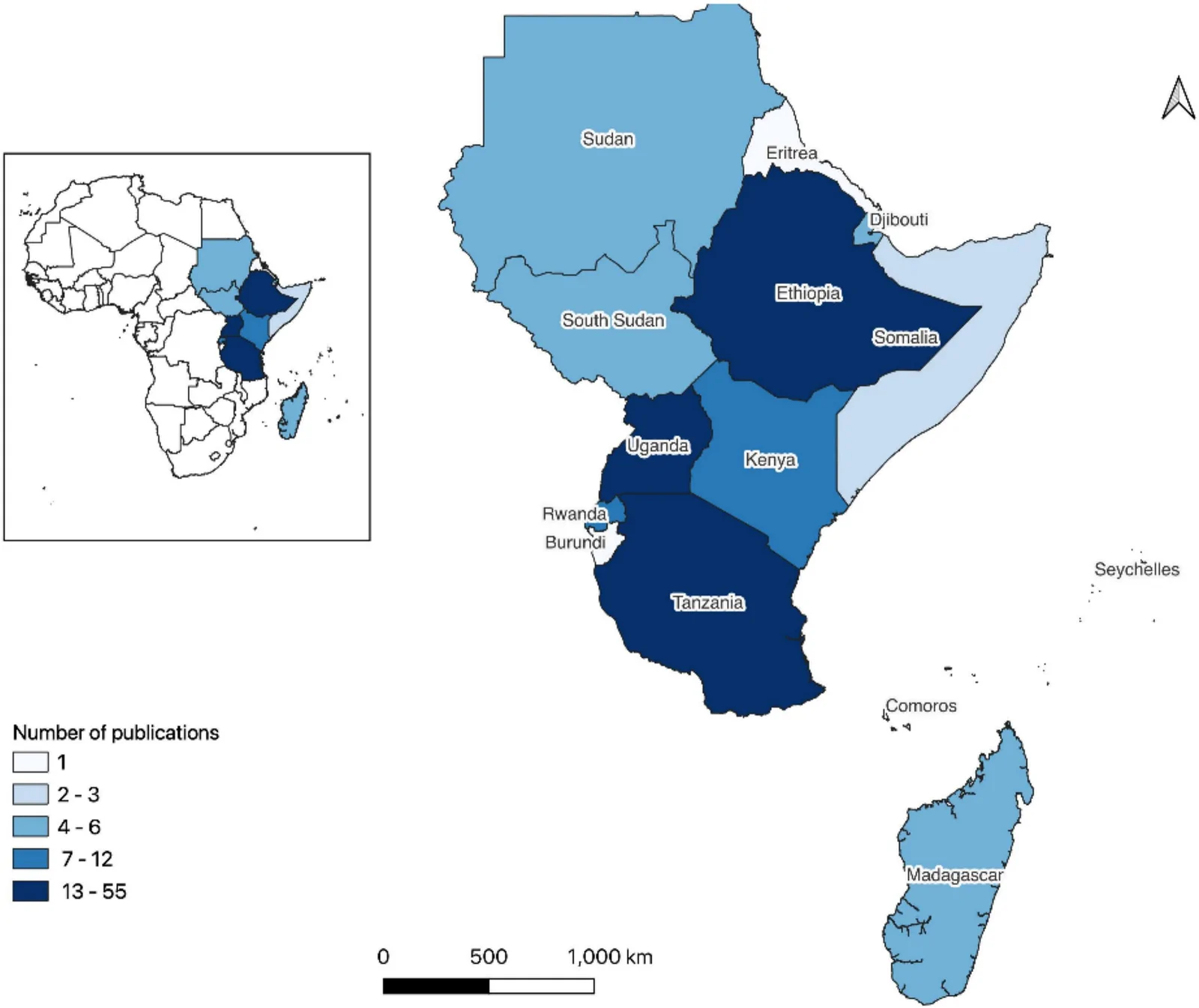
Geographical distribution of the 154 studies included in this review across East African countries. Countries are color-coded by the number of publications, with darker shading representing a greater number of studies. The inset map shows the location of the East African region within the African continent.

### 3.2 Temporal distribution of included studies

Publication output increased markedly over the review period (Fig. 3). Of 154 studies, 15 (9.7%) were published in 2015–2017, 27 (17.5%) in 2018–2020, 63 (40.9%) in 2021–2023 and 49 (31.8%) in 2024–2025. Overall, 112 (72.7%) studies were published from 2021 onward. One health studies showed a similar but more recent increase, with 18 of 19 (94.7%) published since 2021. Ethiopia contributed the most studies, followed by Tanzania and Uganda, while other countries contributed fewer studies intermittently. This pattern indicates rapidly increasing regional research activity, alongside a relatively recent but growing emphasis on multisectoral One Health investigations.

**Fig. 3:**
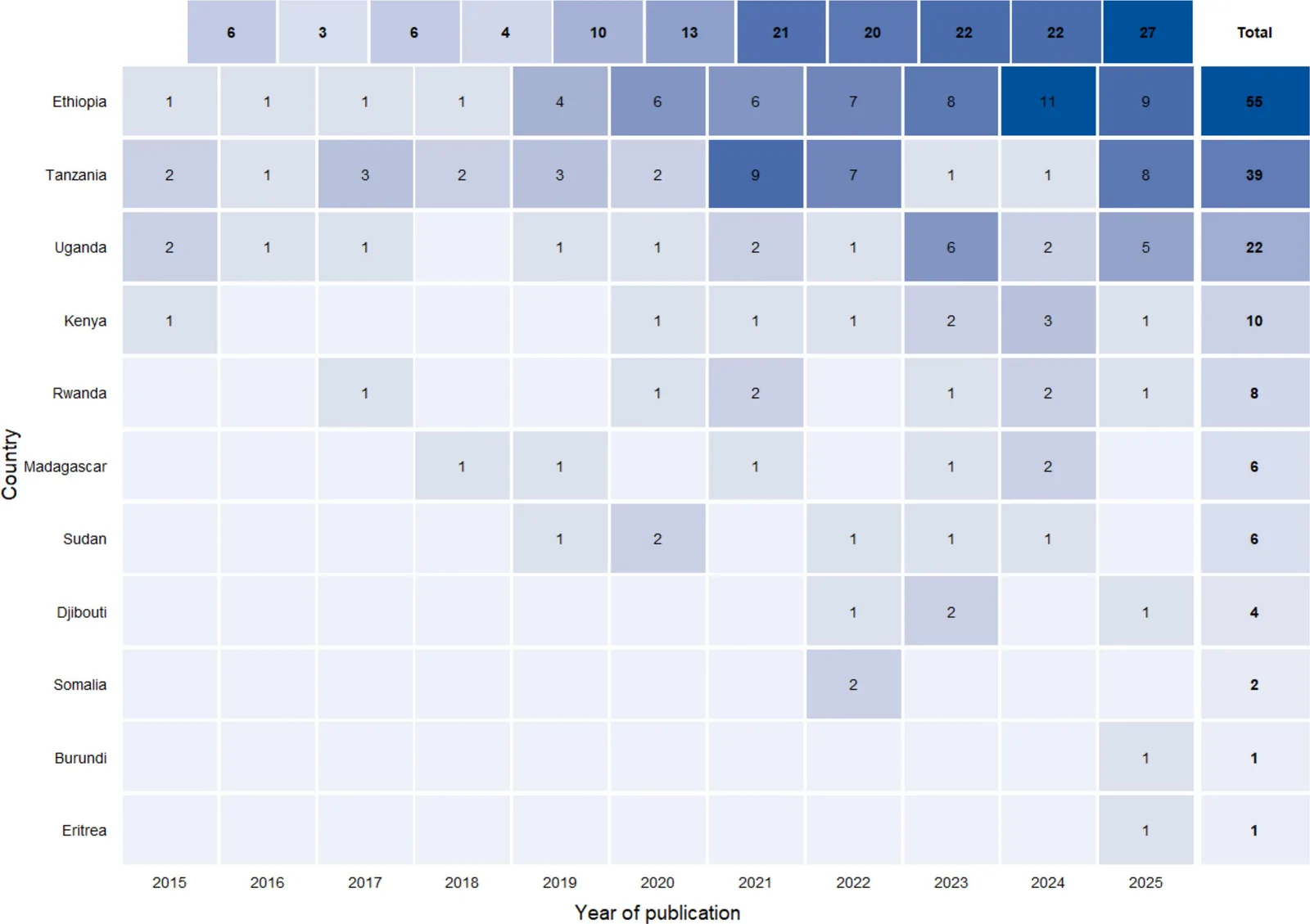
Number of publications by country and year, 2015–2025. Marginal totals show the total publications by year and country.

### 3.3 Distribution and design of One Health interface studies

Among the 154 included studies, 19 (12.3%) employed a One Health, multi-sector sampling design involving two or more interconnected sectors (**Table S3**). Of these, 14 studies investigated all three components of the One Health triad (human, animal and environment), while five examined two-sector interfaces. Studies were conducted across seven countries, mainly Uganda (*n* = 6) and Ethiopia (*n* = 4). Human samples were collected from hospitals and community settings, animal sampling included livestock and companion animals and environmental sampling encompassed soil, drinking water, surface water, wastewater and hospital or household surfaces. Four studies also included food-chain samples, such as dairy products, street-vended foods or market fish. Among 14 studies reporting, sample sizes ranged from 42 to 2508 samples. Whole-genome sequencing was performed in 10 (52.6%) studies, while the others relied on phenotypic AST and/or PCR. ESBL prevalence was reported by 14 (73.7%) studies, carbapenemase prevalence by 9 (47.4%) and both by 8 (42.1%). Four (21.1%) reported neither (**Table S3)**.

### 3.4 Study characteristics, design and sectoral distribution

Within the 154 studies, 74,700 samples were screened, yielding 31,679 bacterial isolates and 21,554 confirmed *E. coli* isolates. Individual study sizes varied widely, ranging from ten samples to >4000 samples. Human-sector specimens predominated, particularly urine, blood, stool or rectal swabs. Animal, food chain and environmental sampling included livestock and wildlife specimens, food products, wastewater, water, soil and surfaces. Human studies accounted for 105 (68%), while 19 (12.3%) adopted a One Health approach spanning two or more sectors; the remainder focused exclusively on the animal 12 (8%), environmental 10 (6%) or food-chain 5(3%) sectors. Cross-sectional designs predominated 132 (86%), followed by cohort 10 (6%), retrospective 7 (5%), surveillance 3 (2%) and case-control 2 (1%) designs. Ethiopia 52 (95%) and Tanzania 34 (87%) showed similar patterns. Other designs were geographically limited, with cohort studies across four countries and surveillance and case-control studies uncommon (Table 1). Advanced genomic characterization was similarly constrained: whole-genome sequencing was performed in only 30 studies (19.5%), while 124 (80.5%) relied on phenotypic and/or PCR-based methods.

**Table 1:** Distribution of study design by country.

Country	Cross-sectional	Cohort	Retrospective	Surveillance	Case-control	Total
Ethiopia	52	2	0	0	1	55
Tanzania	34	4	0	0	1	39
Uganda	16	2	3	1	0	22
Kenya	9	0	0	1	0	10
Rwanda	7	0	1	0	0	8
Sudan	6	0	0	0	0	6
Madagascar	4	2	0	0	0	6
Djibouti	3	0	0	1	0	4
Somalia	0	0	2	0	0	2
Eritrea	0	0	1	0	0	1
Burundi	1	0	0	0	0	1
**Total**	**132**	**10**	**7**	**3**	**2**	**154**

### 3.5 Diagnostic and molecular methods

Culture-based identification was used in all 154 (100%) studies, followed by biochemical testing in 99 (64.3%) studies (Fig. 4). Reported identification methods included culture on non-selective and selective media, Gram staining and biochemical tests such as indole, citrate, urease, and Kligler‑iron-agar or triple-sugar‑iron-based tests. Chromogenic selective media (e.g., CHROMagar ESBL, CHROMagar KPC, CHROMagar ECC) were also reported in some studies for presumptive ESBL/carbapenemase screening or *E. coli* selection. Automated identification systems included MALDI-TOF MS, VITEK 2, API 20E/20NE, BD Phoenix, MicroScan and Microbact. However, methodological details were inconsistently reported and specific culture media and biochemical tests could not be exhaustively tabulated across all 154 studies. This variability in reporting and methodology should be considered when interpreting cross-study comparisons. Phenotypic ESBL detection mainly relied on cephalosporin disk-diffusion screening in 62 (40.3%), combination disk test in 42 (27.3%) and double-disk synergy test in 38 (24.7%). PCR was reported in 57 (37.0%) studies, whereas WGS was reported in 30 (19.5%). MALDI-TOF MS and VITEK 2 were used in 32 (20.8%) and 19 (12.3%), respectively. Carbapenemase detection was predominantly based on carbapenem disk diffusion in 25 (16.2%) and modified carbapenem inactivation method in 13 (8.4%), while multiplex PCR was limited to 11(7.1%) studies.

**Fig. 4:**
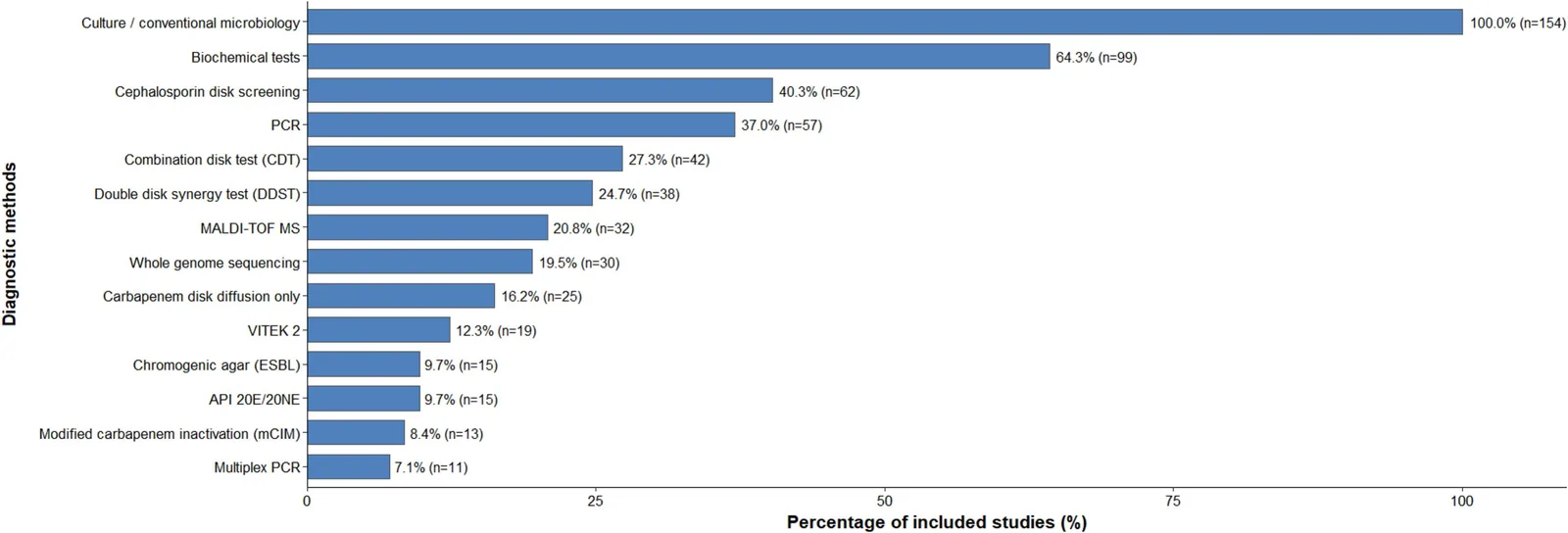
Frequency of diagnostic and molecular methods used across included studies (*n* = 154).

## 4 Distribution, prevalence and molecular epidemiology of ESBL and carbapenemase-producing *E. coli*

### 4.1 Distribution of *E. coli* isolates and ESBL/carbapenemase positivity by country

A total of 21,554 *E. coli* isolates were reported across 11 East African countries. Tanzania contributed the highest number of isolates (6573), followed by Uganda (6263) and Ethiopia (4569). The overall median was 62 isolates per study. Usable ESBL prevalence data were available from 117 studies (76.0%) and carbapenemase prevalence data from 65 (42.2%). Given substantial heterogeneity in populations, settings, diagnostic methods and denominators, prevalence estimates are reported as study-level medians rather than pooled or population-weighted estimates. Tanzania contributed the highest number of ESBL-positive isolates (1982), followed by Ethiopia (1967) and Uganda (1407), while Ethiopia (144) reported the most carbapenemase-positive isolates (Table 2).

**Table 2:** Distribution of *E. coli* isolates and ESBL/carbapenemase-positive isolates by country.

Country	Studies, n (%)	*E. coli* isolates (n)	Median isolates/study	ESBL studies, n	ESBL *E. coli,* n	Carbapenemase studies, n	Carbapenemase *E. coli*, n
Ethiopia	55 (35.7)	4569	35	49	1967	30	144
Tanzania	39(25.3)	6573	72	29	1982	11	33
Uganda	22 (14.3)	6263	114	16	1407	9	140
Kenya	10 (6.5)	1035	99.5	3	77	1	0
Rwanda	8 (5.2)	1176	120	5	755	3	0
Sudan	6 (3.9)	294	38.5	5	113	4	26
Madagascar	6 (3.9)	1019	112	4	930	2	3
Djibouti	4 (2.6)	272	67.5	2	181	3	32
Somalia	2 (1.3)	108	54	2	14	1	0
Eritrea	1 (0.6)	66	66	0	0	0	0
Burundi	1 (0.6)	179	179	1	55	0	0
**Total**	**154 (100)**	**21,554**	**62**	**117**	**7481**	**65**	**378**

Note: Prevalence estimates are study-level medians, not pooled estimates. Country-level estimates based on fewer than 2 studies should be interpreted cautiously.

### 4.2 Prevalence of ESBL and Carbapenemase-producing *E. coli* by sector

Median ESBL prevalence was highest in environmental (71.0%) and human studies (50.0%) and lowest in food-chain studies (23.8%), with an overall median of 46.8%. Carbapenemase prevalence was highest in two-sector human-animal/environment studies (62.3%) and environmental studies (28.6%), with an overall median of 3.3% (Table 3).

**Table 3:** ESBL and carbapenemase prevalence in *E. coli* by sector, reported as median (%) across contributing studies.

Sector	ESBL studies, n	Median ESBL prevalence (%)	Carbapenemase studies, n	Median carbapenemase prevalence (%)
Human	89	50.0	46	3.6
Animal	6	30.3	3	0.0
Environment	5	71.0	3	28.6
Food	3	23.8	2	0.0
One Health (multi-sector)	14	38.2	9	2.5
Human + Animal/Environment (2-sector)	0	–	2	62.3
**Overall**	**117**	**46.8**	**65**	**3.3**

Note: Values are study-level medians, not pooled estimates. Estimates based on fewer than 5 studies should be interpreted cautiously.

### 4.3 Temporal trends in ESBL and Carbapenemase prevalence

Median ESBL prevalence fluctuated, rising from 38.0% in 2015–2017 to 39.7% in 2018–2020 and peaking at 50.4% in 2021–2023, before declining to 42.8% in 2024–2025. In contrast, median carbapenemase prevalence rose steadily from 1.8% in 2015–2017 to 7.5% in 2024–2025 (**Table S4**; Fig. 5). However, differences in contributing countries, populations, settings and diagnostic/molecular methods indicate that this pattern represents variation in study-level prevalence across publication periods rather than a confirmed regional temporal increase in carbapenemase-producing *E. coli*.

**Fig. 5:**
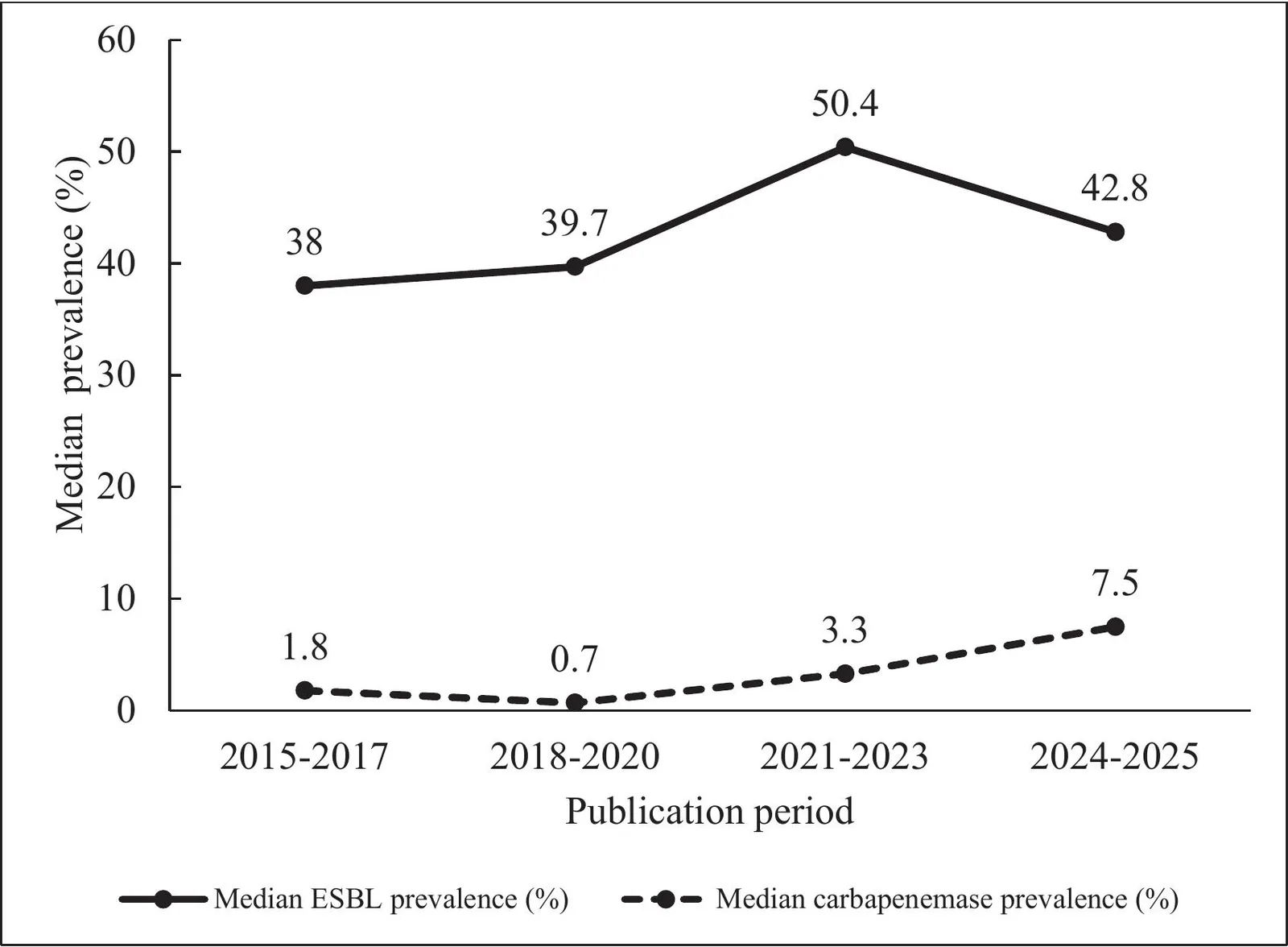
Temporal trend in median ESBL and carbapenemase prevalence in *E. coli* by publication period, East Africa (2015–2025). Solid line represents median ESBL prevalence (%); dashed line represents median carbapenemase prevalence (%) across studies reporting each outcome within each publication period.

### 4.4 Phenotypic antimicrobial resistance profile of *E. coli* isolates

Phenotypic (AST) covered 25 antibiotics across 12 classes (**Table S11**). Median resistance was highest for amoxicillin (97.5%), ampicillin (90.0%) and aztreonam (85.3%), and remained substantial for third-generation cephalosporins (cefotaxime 52.9%, ceftazidime 47.9%, ceftriaxone 46.8%), consistent with the high ESBL prevalence reported above. Ciprofloxacin (37.2%) and gentamicin (31.2%) resistance were intermediate, while resistance remained low for colistin (0%), imipenem (2.3%), ertapenem (2.9%), meropenem (4.3%), fosfomycin (4.7%), and amikacin (6.0%), with considerable inter-study heterogeneity observed.

## 5 Molecular characterization and epidemiology of antimicrobial resistance

Table S12 summarizes the number of studies contributing to each molecular analysis reported in this section.

### 5.1 Distribution of *E. coli* ESBL and carbapenemase genes

Molecular gene detection was reported in a smaller subset of studies than phenotypic resistance. ESBL genes were characterised in 69 (44.8%) of 154 studies, compared with 117 (76.0%) reporting phenotypic ESBL prevalence, while carbapenemase genes were characterised in 40 (26.0%), compared with 65 (42.2%) reporting phenotypic carbapenemase prevalence. Detection of resistance genes does not necessarily indicate phenotypic resistance; therefore, molecular and phenotypic findings were considered separately.

Among 69 studies reporting ESBL genes, *bla*_CTX-M_ was common in 45 (65.2%), followed by *bla*_TEM_, 34 (49.3%) and *bla*_SHV,_ 25 (36.2%). *bla*_CTX-M-15_ was the predominant variant 18 (26.1%), followed by *bla*_CTX-M-27_, 9 (13.0%); *bla*_OXA_ and *bla*_OXA-1_ occurred in 17.4% and 15.9% of studies, respectively (Fig. 6). The *bla*_CTX-M_ family predominated across most countries, reported in all gene-tested studies from Burundi, Djibouti and Sudan and most studies from Ethiopia, Kenya, Rwanda and Tanzania (**Fig. S1**). The *bla*_TEM_ family was also widely distributed, particularly in Burundi, Djibouti, Kenya and Uganda. Similarly, *bla*_SHV_ was frequent in Burundi, Kenya and Ethiopia. *bla*_OXA_ genes were infrequently reported across the region. *bla*_CTX-M-15_ was particularly common in Madagascar 4 (50.0%) and Tanzania 24 (37.5%), whereas *bla*_CTX-M-27_ was less common, occurring in Madagascar 4 (25.0%), Tanzania 24 (16.7%), Ethiopia 14 (14.3%) and Uganda 11(9.1%). Estimates from countries with few gene-tested studies should be interpreted with caution.

**Fig. 6:**
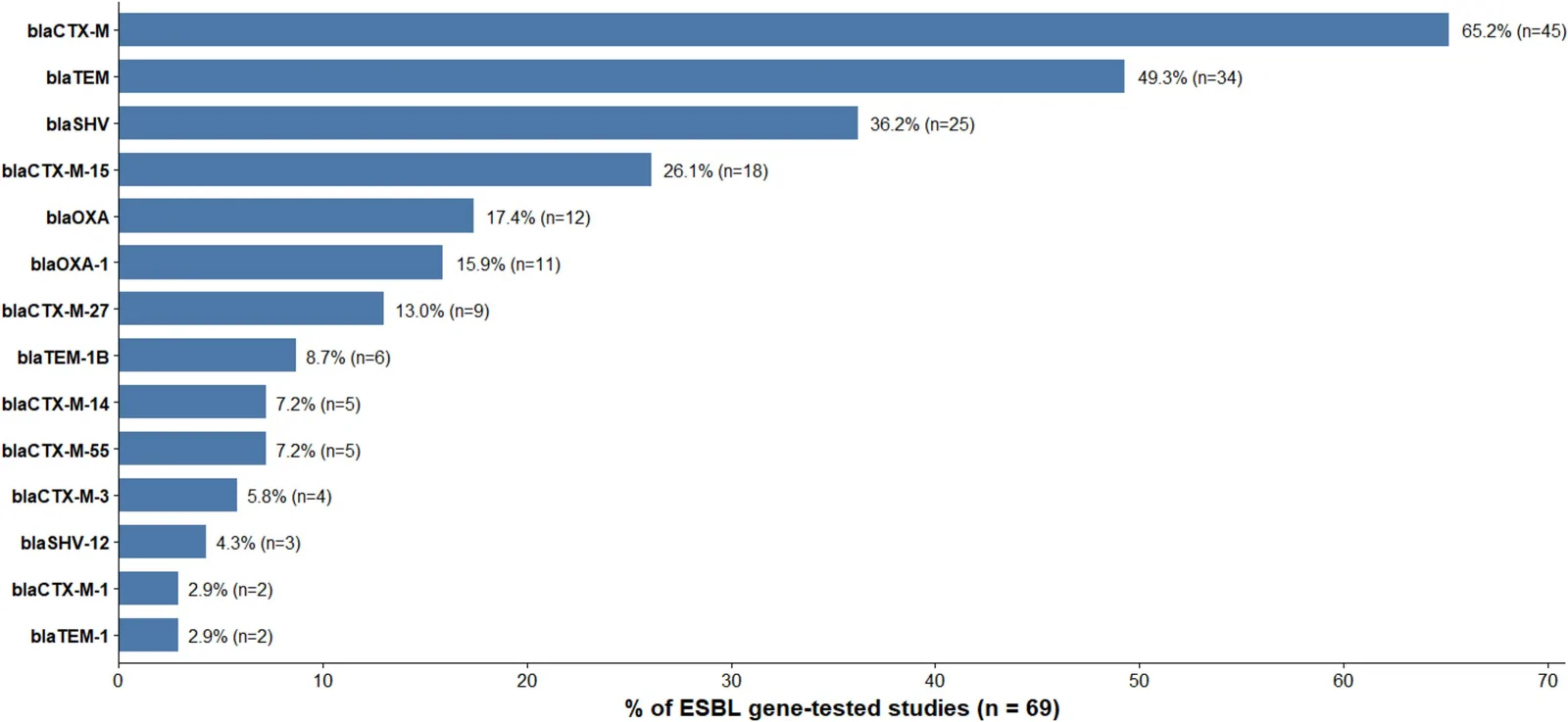
Distribution of ESBL genes and variants detected in *E. coli* isolates among ESBL gene-tested studies (*n* = 69).

Among the 40 studies investigating carbapenemase genes, *bla*_NDM_ was the most frequently reported gene family 11 (27.5%), followed by *bla*_KPC_ and *bla*_OXA-48,_ 8 each (20.0%) (Fig. 7). *bla*_NDM-5_ 5 (12.5%), *bla*_IMP_ and *bla*_OXA-48,_ each 4 (10.0%) were also reported.*bla*_NDM_ predominated in Ethiopia (50.0%), followed by Djibouti (25.0%), Uganda (22.2%) and Tanzania (16.7%) as illustrated in **Fig. S2**. Overall, *bla*_NDM_, *bla*_KPC_ and *bla*_OXA-48/OXA-48-like_ were the predominant carbapenemase gene families circulating in East Africa, although evidence remains limited outside Ethiopia and Uganda. Estimates from countries with few gene-tested studies should be interpreted with caution.

**Fig. 7:**
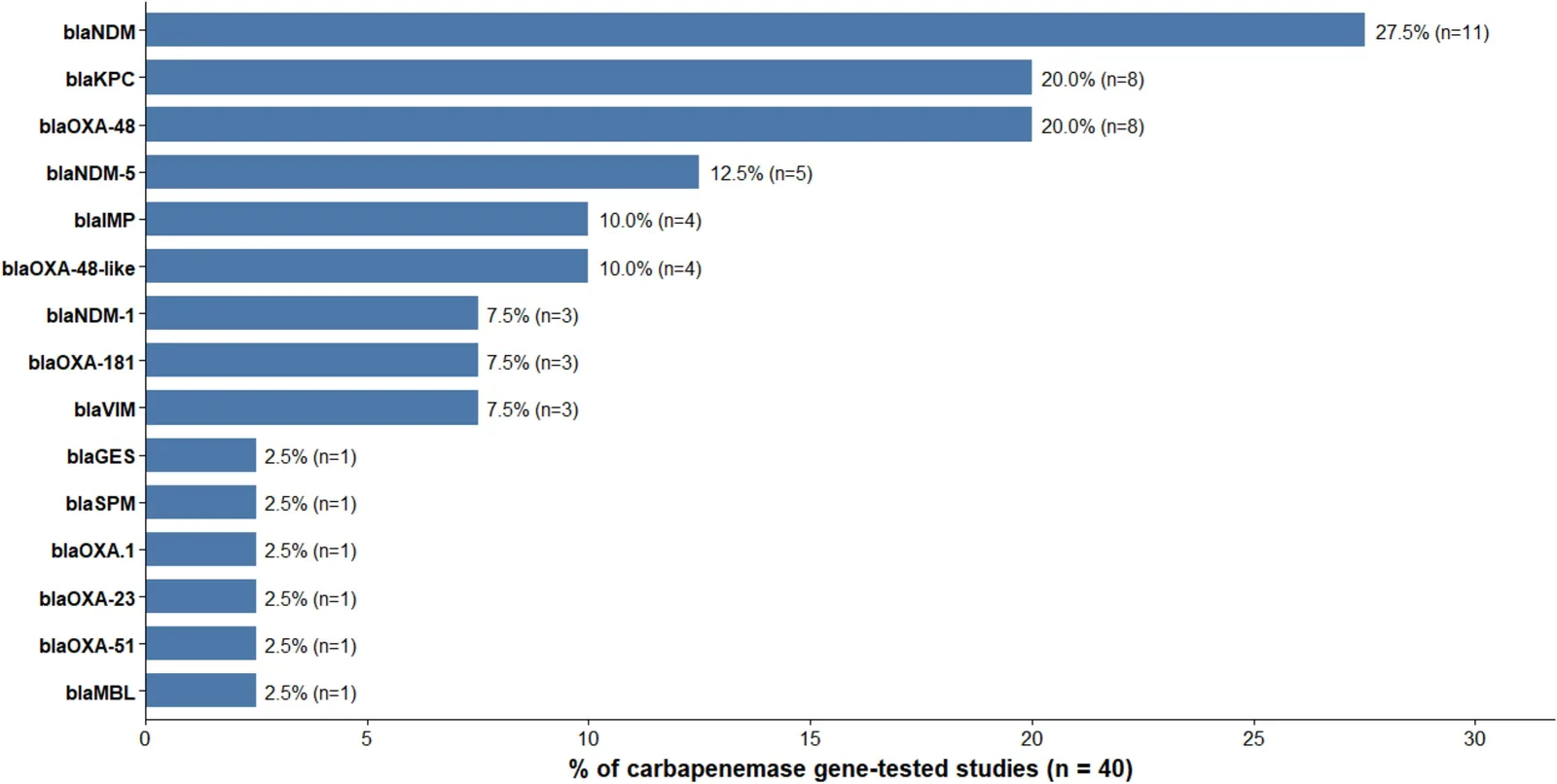
Distribution of carbapenemase genes and variants detected in *E. coli* isolates among carbapenemase gene-tested studies (*n* = 40).

## 6 Multi-locus sequence typing and cross-sectoral distribution of *E. coli* sequence types

Multi-locus sequence typing (MLST) was reported in 31 studies (20.1%), identifying 120 distinct sequence types (STs). ST131 was most frequent 17 (54.8%), followed by ST10, ST38 and ST410 (13 each, 41.9%) and ST167 (10,32.3%), full ST ranking **Table S5**.

A total of 118 distinct sequence types (STs) were distributed across the human, animal, and environmental sectors (Fig. 8). Nearly half of the identified STs, 48 (40.7%), shared across human, animal and environmental sources. Human isolates showed the greatest ST diversity, while animal and environment specific STs were fewer. ST identities for Fig. 8 are provided in **Table S6**.

**Fig. 8:**
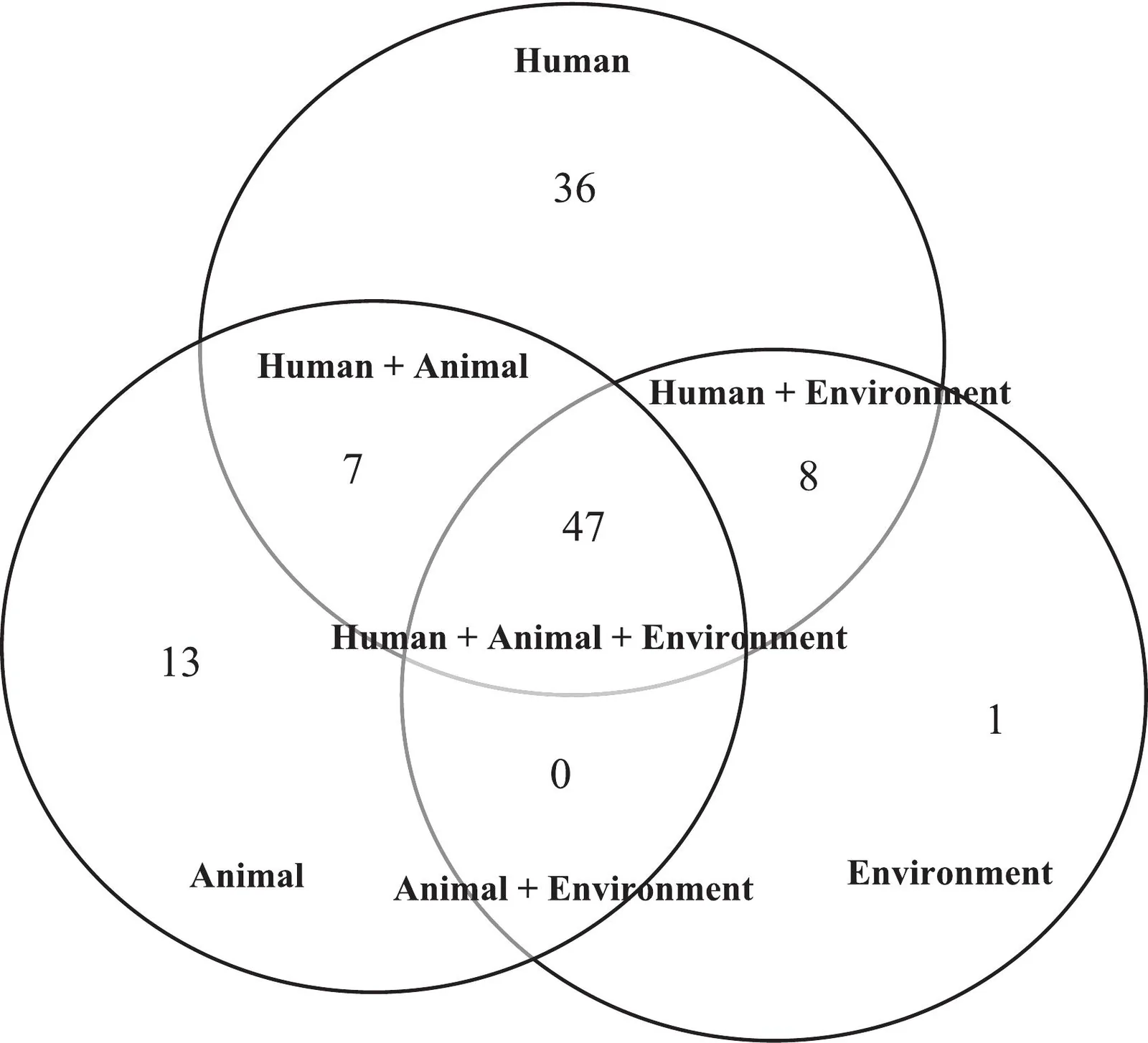
Venn diagram showing the number of *E. coli* sequence types (STs) across human, animal and environmental sectors, East Africa. Shared STs do not demonstrate transmission.

### 6.1 Country-level distribution of predominant sequence types

The distribution of predominant sequence types varied across the 31 Multi-locus sequence typing (MLST)-tested studies (**Fig. S3**). ST131 was most frequent in Tanzania 7 (70.0%), 4 (66.7%) from Ethiopia and 50.0% of studies from Uganda, Madagascar and Rwanda. ST410 predominated in Djibouti 2 (100.0%) and Ethiopia 5 (83.3%). ST10 was reported in 50.0% of studies from Rwanda, Djibouti, Madagascar, Ethiopia and Uganda and 30.0% from Tanzania. ST38 predominated in Rwanda (100.0%), followed by Ethiopia, Madagascar and Djibouti (50.0% each). ST167 (58.0%) and ST617 (40.0%) were most common in Tanzania, while ST48 occurred in Kenya's single MLST-tested study. Findings from countries with few MLST-tested studies should be interpreted cautiously.

#### 6.1.1 Phylogroups

Clermont phylogrouping was reported in 22 studies. Overall, phylogroup A was reported in 19(86.4%) and B1, 17 (77.3%) most frequently identified, followed by B2 and D in 13 (59.1%) of studies. Phylogroups C and F were each detected in 12 (54.5%), while phylogroups E, G and Clade I were less common (31.8%, 13.6% and 13.6%, respectively) (**Table S7**).

Distribution varied geographically (**Fig. S4)**, with phylogroup A widely reported across countries and B1 broadly distributed. The extraintestinal pathogenic phylogroups B2 and D were commonly reported in Ethiopia (75.0% each), while phylogroup D was identified in all phylotyped studies from Uganda and Djibouti. Most countries had few phylotyped studies, limiting national prevalence estimates.

#### 6.1.2 Plasmid replicon typing

Plasmid replicon typing was reported in 21 studies, identifying 53 unique plasmid replicon types (**Table S8**). IncF-family replicons were the most frequently reported, with IncFIA and IncFII each detected in 12 (57.1%) of replicon-typed studies, followed by IncFIB and IncY, each detected in 8 (38.1%). Col (BS512), IncI1 and IncQ1 were each identified in 6 (28.6%) of studies.

#### 6.1.3 Virulence genes

Virulence gene profiling was performed in 25 studies (16.2%), identifying 256 distinct virulence-associated genes. The most frequently detected were *iss* 9 (36.0%), *gad*, *lpfA*, and *traT*, 8 each (32.0%) and *iutA,* 7 (28.0%), spanning serum resistance, adhesion, and iron-acquisition functions. (**Table S9**).

#### 6.1.4 Inferred transmission pathways

Of the 154 studies, 71 (46.1%) reported at least one inferred transmission pathway. Nosocomial transmission was most frequent 32 (45.1%), followed by environmental 27 (38.0%), community 26 (36.6%), zoonotic 22 (31.0%), food chain 12 (16.9%), waterborne 12 (16.9%) and human-to-human transmission 9 (12.7%). Nosocomial transmission predominated in Ethiopia 15 (46.9%) and Tanzania 10 (31.3%), whereas environmental transmission was most commonly reported in Uganda 10 (37.0%). Zoonotic transmission was most common in Ethiopia 8 (36.4%) and Uganda 6 (27.3%) (Fig. 9).

**Fig. 9:**
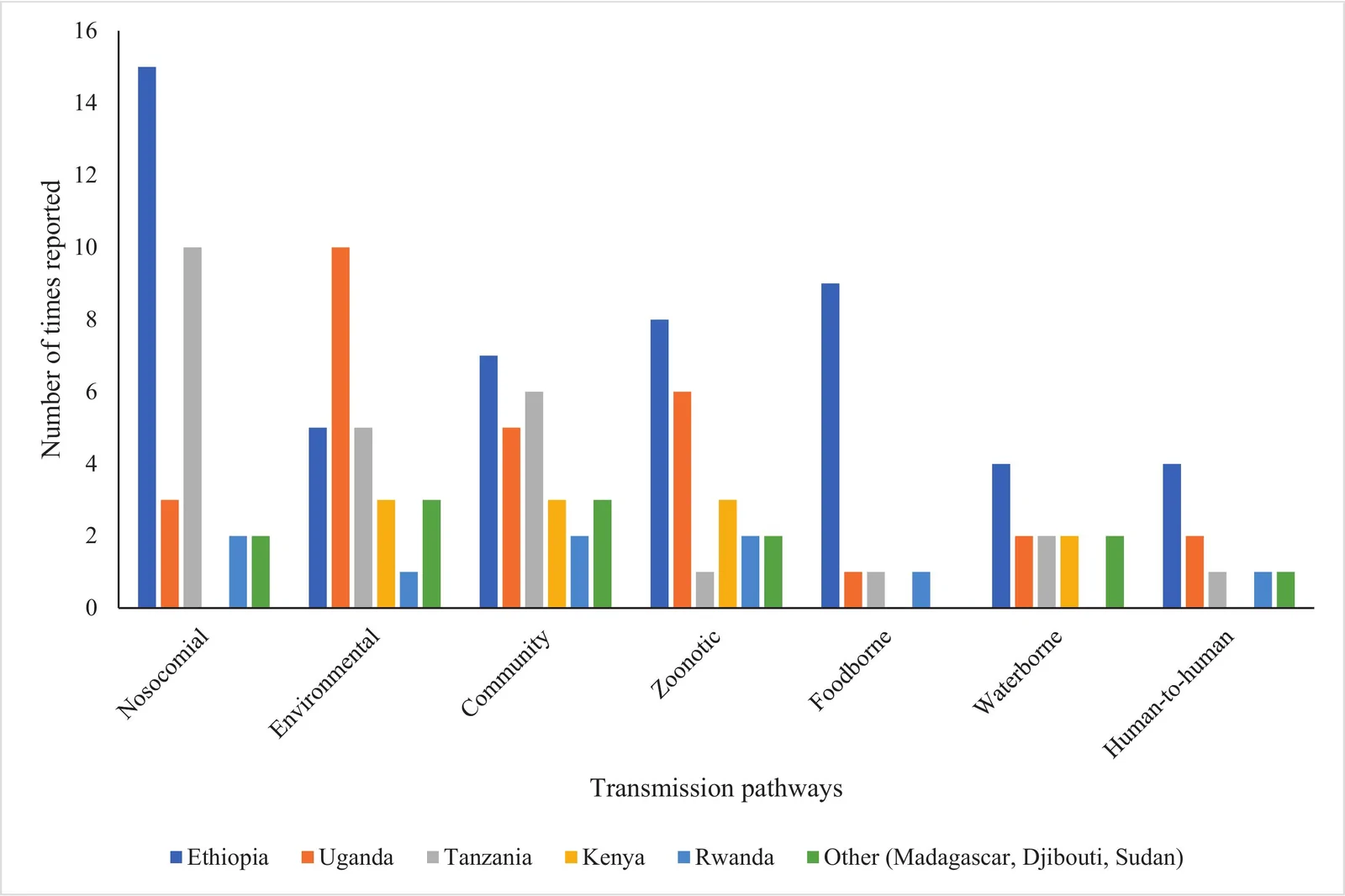
Inferred transmission pathways of ESBL/carbapenemase-producing *E. coli* by country, East Africa.

#### 6.1.5 One Health transmission evidence

Among 19 One Health studies, 14 (73.7%) included all three sectors, while five (26.3%) investigated only two sectors. WGS provided molecular evidence of cross-sector transmission in 10 (52.6%) studies, based on shared sequence types, resistance genes and plasmids. Evidence from Rwanda, Madagascar and Djibouti indicated bidirectional transmission across sectors. In the large Malawi and Uganda genomic study, inferred human-environment transmission events (*n* = 463) exceeded human-animal (*n* = 146) or animal-environment (*n* = 142) events, underscoring the environment as a major transmission compartment. The remaining studies inferred transmission using phenotypic AST profiles, household co-occurrence of resistant isolates or epidemiological associations. One Ugandan study found 67% of cattle isolates closely related to human isolates, while another found no association between livestock ownership and resistant *E. coli* carriage. Detailed evidence is provided in Table 5.

**Table 5:** Cross-sectoral transmission evidence in One Health studies, by method.

Study	Country	Method	Shared STs/genes/plasmids	Transmission evidence
Eger 2021 [14]	Rwanda	WGS	ST131, ST648, ST354, ST38; *bla*_CTX-M-15_; IncF	Near-identical SNP distances across patients, caregivers, animals, housefly
Gay 2023 [15]	Madagascar	WGS	84 STs (ST3489 dominant); *bla*_CTX-M-15_; IncF, IncY	47.1% of 104 transmission clusters spanned human + animal hosts
Mohamed 2023 [19]	Djibouti	WGS	ST10; *bla*_OXA-181_; ColKP3-IncX3	Clonal + plasmid spread across humans, livestock, fish, wastewater
Musicha 2025 [20]	Uganda	WGS	ST10; *bla*_CTX-M-15/27_, *bla*_NDM-5_	Human-environment transmission events (*n* = 463) more frequent than human-animal (*n* = 146) or animal-environment (*n* = 142) events
Milenkov 2024 [21]	Madagascar	WGS	ST10; *bla*_CTX-M-15_; IncF, IncY	Core-genome SNP clustering across human/chicken/environment
Geuther 2023 [22]	Rwanda	Phenotypic only	–	No association between livestock ownership and human carriage
Muleme 2023 [23]	Uganda	Phenotypic only	–	Shared antibiogram patterns across households (no molecular confirmation)
Kavulavu 2025 [24]	Kenya	Phenotypic only	–	Household co-positivity across human, animal, and environmental samples
Iramiot 2020 [25]	Uganda	WGS	phylogroups A/B1/B2/D; *bla*_OXA-1_, *bla*_TEM-1_	67% of cattle isolates closely related to human isolates, but limited virulence-gene overlap (no simple zoonotic spillover)

#### 6.1.6 Risk factors

Risk factor analysis was reported in 113 (73.4%) studies. Prior antibiotic use was the most consistently associated factor, followed by healthcare exposure including prior hospitalization, catheterization, Intensive and neonatal intensive care unit (ICU/NICU) admission and use of invasive devices. Young age among children and neonates was also frequently associated with carriage or infection. One Health studies highlighted animal contact, untreated water sources and poor sanitation or hygiene. Some studies found no significant associations, including livestock ownership (**Table S10**).

## 7 Discussion

Overall, longitudinal *E. coli* genomic surveillance data relevant to tracking emerging MDR strains remain limited in East Africa. Nevertheless, this review demonstrates that ESBL-producing *Escherichia coli* is firmly established across East Africa. Carbapenemase-producing lineages remain comparatively uncommon, with higher median prevalence in recent publication periods, consistent with global forecasts of rising carbapenem resistance. Whether this reflects true emergence or differences in surveillance and study composition remains unclear given the limited availability of longitudinal genomics data in humans, animals, food and the environment [3].

*bla*_CTX-M-15_ was the predominant ESBL determinant, while sequence types ST131, ST10, ST38 and ST410 represented the major high-risk clones, consistent with the globally recognized epidemiology of extraintestinal pathogenic *E. coli* [26]. Notably, nearly half of the identified STs reported across more than one human, animal and environmental sector, suggesting circulation of shared lineages across sectors. However, shared STs alone do not establish transmission, directionality or epidemiological linkage, as most originated from independent studies. These findings support the need for coordinated One Health interventions rather than sector-specific control strategies [6].

These findings highlight the importance of a One Health approach to understand the epidemiology of resistant *E. coli*. Resistant *E. coli* can disseminate across sectors through direct animal contact, contaminated food and polluted water [14], [27], [28]. Despite conditions in the region that promote such dissemination, a major gap is that only 12.3% of *E. coli* studies adopted a clear One Health design. The consistently high ESBL burden alongside occasional carbapenemase data mirrors the broader pattern of an entrenched ESBL epidemic in SSA, with an emerging carbapenemase threat [28]. Most studies were from Ethiopia, Tanzania and Uganda, likely reflecting differences in laboratory capacity and research investment rather than true AMR variation.

Burundi, Eritrea and Somalia remained major surveillance blind spots, and Comoros, Mauritius, Seychelles and South Sudan contributed no eligible studies, consistent with the concentration of African AMR genomic surveillance in few well-resourced hubs [29].

This geographic skew suggests that reported prevalence may reflect research capacity as much as regional epidemiology. The widespread occurrence of *bla*_CTX-M-15_ confirms its continued dominance as the principal ESBL determinant across East Africa, consistent with reports from elsewhere, while the co-detection of *bla*_NDM_ and *bla*_OXA-48_ like genes is hypothetically consistent with a gradual emergence of carbapenem resistance in the region [28], [30].

The discrepancy between phenotypic carbapenem resistance and carbapenemase gene detection, may reflect limited carbapenem susceptibility testing and suggests that phenotypic screening alone could underestimate carbapenemase dissemination. Furthermore, variation in dominant ESBL and carbapenemase genes between countries, including *bla*_CTX-M_ versus *bla*_TEM_ predominance and distinct *bla*_NDM_ versus *bla*_OXA-48_ further demonstrates substantial regional heterogeneity. Similar national-level variation reported in West Africa [31] suggests that country-specific resistance gene dominance may represent a broader African pattern.

Globally disseminated high-risk clones (ST131, ST10, ST38 and ST410) occurred across multiple East African countries, suggesting the influence of internationally successful lineages. This aligns with genomic findings from Uganda [32], with reports elsewhere describing these lineages as major contributors of ESBL dissemination, though the descriptive, largely non-genomically linked design of most included studies limits causal inference about the specific drivers of their spread in East Africa.

The predominance of phylogroups A/B1, alongside substantial B2/D representation, aligns with the distribution of high-risk lineages, particularly ST131 and widespread *bla*_CTX-M-15._

IncF-family plasmids (IncFIA/IncFII) were the most frequently identified replicons among replicon-typed studies, while *bla*_CTX-M-15_ was the most frequently detected ESBL variant among gene-tested studies. Direct genomic evidence that *bla*_CTX-M-15_ carried on an IncF plasmid within the same isolate was confirmed only in whole-genome-sequenced studies [14], [15], [21], compatible with, but not establishing, horizontal gene transfer as a major driver of cross-sectoral dissemination region-wide, alongside clonal expansion of high-risk lineages.

Whole-genome sequencing was used in only a minority of One Health studies, with genomic confirmation of cross-sector transmission achieved in about half. Consequently, many inferred transmission pathways relied on epidemiological associations rather than genomic evidence. This reflects broader limitations, including cross-sectional designs, non-probability sampling and limited high-resolution genomics for establishing directional transmission [29].

Nosocomial and environmental pathways predominated in our review, contrasting with a continent-wide scoping review identifying the food chain as the leading transmission route [33]. This divergence likely reflects study composition: our evidence base dominated by hospital-based human studies, whereas Nnah et al.'s [33] emphasized the farm-to-fork continuum, rather than a genuine regional epidemiological difference, with real policy implications. The apparent nosocomial predominance we found may partly reflect the study designs in East Africa that had limited evaluation of foodborne transmission, rather than the absence of this AMR transmission pathway in the region.

Risk factors for carriage differed systematically by sector, with prior antibiotic use, healthcare exposure and young age predominating in human clinical studies, aligning with established global evidence [34]. In contrast, One Health studies more frequently implicated animal contact, unsafe water and poor sanitation, reinforcing water, sanitation, and hygiene (WASH) deficiencies as a key cross-sectoral driver often missed by clinical surveillance. Null risk-factor findings may reflect widespread background carriage. These findings underscore the need for integrated AMR interventions that combine antimicrobial stewardship with WASH improvements, environmental management and improved animal husbandry.

The main strength of this review is its comprehensive synthesis of evidence on ESBL and carbapenemase-producing *Escherichia coli* across the human, animal and environmental sectors, integrating molecular epidemiology, resistance genes, high-risk clones, plasmid replicons, transmission pathways and risk factors. Several limitations should be considered. First, restricting the search to PubMed, Scopus and English-language publications may have excluded relevant non-English, regional and grey literature, potentially under-representing some East African countries. Second, substantial methodological heterogeneity limited direct comparison across studies [35]. Third, WGS was used in few studies, restricting high-resolution assessment of resistance and transmission [29]. Fourth, the geographic and sectoral coverage was uneven, with evidence concentrated in Ethiopia, Tanzania and Uganda and predominantly based on human clinical isolates and phenotypic methods. Finally, most transmission pathways were epidemiologically inferred rather than genomically demonstrated and should therefore be interpreted cautiously. Similarly, the apparent rise in reported prevalence over time is difficult to interpret nationally and regionally because of the heterogeneity in study designs and genotyping methodologies, as well as the under representation of some countries in the region.

### 7.1 Implications

These findings highlight the need to strengthen integrated One Health surveillance across East Africa through expanded genomic sequencing capacity particularly in underrepresented countries, standardized carbapenemase testing and harmonized laboratory methods. AMR control should combine antimicrobial stewardship, infection prevention and WASH interventions across sectors. The geographical imbalance, methodological heterogeneity and limited WGS limits interpretation of these genomics data to strengthen surveillance. Future research should prioritize longitudinal, genomically informed studies to clarify transmission pathways of MDR *E. coli* across the human-animal-environment interface, thereby informing appropriate and context-specific AMR control interventions.

## 8 Conclusion

This scoping review maps evidence of ESBL and carbapenemase-producing *Escherichia coli* across human, animal and environmental sectors in East Africa. ESBL-producing *E. coli* was widely reported, with *bla*_CTX-M-15_ and IncF family plasmid replicons among the most frequently detected determinants, though direct plasmid-gene linkage was genomically confirmed in only a small subset of studies alongside high-risk lineages including ST131, ST10, ST38 and ST410. Carbapenemase-producing strains carrying *bla*_NDM_ and *bla*_OXA-48_-like genes remain less widespread. We recommend strengthening genomic capacity to improve surveillance, standardized diagnostics, antimicrobial stewardship and WASH interventions to control MDR pathogens, including ESBL and carbapenemase-producing *Escherichia coli* in the region.

## Use of AI or Large language models

AI was used for language editing and manuscript formatting. All scientific content, analyses, interpretations, and conclusions were developed by the authors.

## CRediT authorship contribution statement

**Magreth Erick Macha:** Writing – review & editing, Writing – original draft, Investigation, Formal analysis, Data curation, Conceptualization. **Alisen Ayitewala:** Writing – review & editing, Methodology, Investigation, Conceptualization. **Damalie Nakanjako:** Writing – review & editing, Supervision, Project administration, Formal analysis, Data curation, Conceptualization.

## Consent for publication

Not available.

## Ethics approval and consent to participate

Not applicable. This scoping review used only previously published data and did not involve human participants or the collection of new data.

## Funding

This study was funded by the African Research Universities Alliance (ARUA)-Mastercard Foundation/Carnegie Fellowship that funded Dr. Magreth Erick Macha placement at the Infectious Diseases Institute's Translational laboratory at Makerere University and the Research Network for Health Innovations in Sub-Saharan Africa (RHISSA), a funding initiative of the German Federal Ministry of Research, Technology and Space (BMFTR) under the ADAPT One Health Network project, grant agreement number 81300905, that supported Mr. Alisen Ayitewala and Prof. Damalie Nakanjako. The funders had no role in the study design, data collection, data analysis, data interpretation, manuscript writing, or the decision to submit the manuscript for publication.

## Declaration of competing interest

We declare no competing interests.

## Data Availability

All data generated or analyzed during this study are included in this published article and its Supplementary information files.

## Appendix A Supplementary data

Table S1. Electronic database and search strings used to identify studies on ESBL and carbapenemase-producing *E. coli* in East Africa (01/2015-12/2025) for the scoping review Table S2. Data variables and extraction parameters Table S3. Characteristics of One Health studies (n = 19) across human, animal, food and environmental sectors Table S4. Temporal trend in median ESBL and carbapenemase prevalence in E. coli by publication period Table S5. Predominant *E. coli* sequence types identified across MLST-tested studies (n=31) Table S6. Distribution of *Escherichia coli* sequence types (STs) across One Health sector combinations. Table S7. Distribution of *E. coli* phylogroups across phylotyped studies (n=22) Table S8. Predominant plasmid replicon types identified across replicon-typed studies (n=21) Table S9. Predominant virulence genes identified across virulence-tested studies (n=25) Table S10. Risk factors investigated and identified, organized by category and sector Table S11. Antimicrobial resistance phenotype of *E. coli* isolates by antibiotic (n=25) Table S12. Number of studies contributing to each molecular/genomic analysis (n = 154) Figure S1 Country-level heatmap; cell values indicate the percentage of gene-tested studies per country reporting each gene, with darker shading representing higher frequencies. Figure S2 Country-level heatmap of carbapenemase gene distribution in *E. coli*; cell values indicate the percentage of gene-tested studies per country reporting each gene, with darker shading representing higher frequencies. Figure S3 Heatmap showing the distribution of predominant *E. coli* sequence types (STs) across East African countries. Cell values indicate the percentage of MLST-tested studies reporting each ST, with the number of MLST-tested studies shown beside each country (n). Darker blue shading represents higher reporting frequencies. Figure S4 Heatmap showing the distribution of *E. coli* Clermont phylogroups across East African countries. Cell values represent the percentage of phylogroup-tested studies reporting each phylogroup, with the number of phylogroup-tested studies indicated in parentheses beside each country.

## References

[1] Murray C.J., Ikuta K.S., Sharara F., Swetschinski L., Robles Aguilar G., Gray A., Han C., Bisignano C., Rao P., Wool E., Johnson S.C., Browne A.J., Chipeta M.G., Fell F., Hackett S., Haines-Woodhouse G., Kashef Hamadani B.H., Kumaran E.A.P., McManigal B., Agarwal R., Akech S., Albertson S., Amuasi J., Andrews J., Aravkin A., Ashley E., Bailey F., Baker S., Basnyat B., Bekker A., Bender R., Bethou A., Bielicki J., Boonkasidecha S., Bukosia J., Carvalheiro C., Castañeda-Orjuela C., Chansamouth V., Chaurasia S., Chiurchiù S., Chowdhury F., Cook A.J., Cooper B., Cressey T.R., Criollo-Mora E., Cunningham M., Darboe S., Day N.P.J., De Luca M., Dokova K., Dramowski A., Dunachie S.J., Eckmanns T., Eibach D., Emami A., Feasey N., Fisher-Pearson N., Forrest K., Garrett D., Gastmeier P., Giref A.Z., Greer R.C., Gupta V., Haller S., Haselbeck A., Hay S.I., Holm M., Hopkins S., Iregbu K.C., Jacobs J., Jarovsky D., Javanmardi F., Khorana M., Kissoon N., Kobeissi E., Kostyanev T., Krapp F., Krumkamp R., Kumar A., Kyu H.H., Lim C., Limmathurotsakul D., Loftus M.J., Lunn M., Ma J., Mturi N., Munera-Huertas T., Musicha P., Mussi-Pinhata M.M., Nakamura T., Nanavati R., Nangia S., Newton P., Ngoun C., Novotney A., Nwakanma D., Obiero C.W., Olivas-Martinez A., Olliaro P., Ooko E., Ortiz-Brizuela E., Peleg A.Y., Perrone C., Plakkal N., Ponce-de-Leon A., Raad M., Ramdin T., Riddell A., Roberts T., Robotham J.V., Roca A., Rudd K.E., Russell N., Schnall J., Scott J.A.G., Shivamallappa M., Sifuentes-Osornio J., Steenkeste N., Stewardson A.J., Stoeva T., Tasak N., Thaiprakong A., Thwaites G., Turner C., Turner P., van Doorn H.R., Velaphi S., Vongpradith A., Vu H., Walsh T., Waner S., Wangrangsimakul T., Wozniak T., Zheng P., Sartorius B., Lopez A.D., Stergachis A., Moore C., Dolecek C., Naghavi M. (2022). Global burden of bacterial antimicrobial resistance in 2019: a systematic analysis. Lancet.

[2] Sartorius B., Gray A.P., Weaver N. Davis, Aguilar G. Robles, Swetschinski L.R., Ikuta K.S., Mestrovic T., Chung E., Wool E.E., Han C., Hayoon A. Gershberg, Araki D.T., Abd-Elsalam S., Aboagye R.G., Adamu L.H., Adepoju A.V., Ahmed A., Akalu G.T., Akande-Sholabi W., Amuasi J.H., Amusa G.A., Argaw A.M., Aruleba R.T., Awoke T., Ayalew M.K., Azzam A.Y., Babin F.-X., Banerjee I., Basiru A., Bayileyegn N.S., Belete M.A., Berkley J.A., Bielicki J.A., Dekker D., Demeke D., Demsie D.G., Dessie A.M., Dunachie S.J., Ed-Dra A., Ekholuenetale M., Ekundayo T.C., El Sayed I., Elhadi M., Elsohaby I., Eyre D., Fagbamigbe A.F., Feasey N.A., Fekadu G., Fell F., Forrest K.M., Gebrehiwot M., Gezae K.E., Ghazy R.M., Hailegiyorgis T.T., Haines-Woodhouse G., Hasaballah A.I., Haselbeck A.H., Hsia Y., Iradukunda A., Iregbu K.C., Iwu C.C.D., Iwu-Jaja C.J., Iyasu A.N., Jaiteh F., Jeon H., Joshua C.E., Kassa G.G., Katoto P.D.M.C., Krumkamp R., Kumaran E.A.P., Kyu H.H., Manilal A., Marks F., May J., McLaughlin S.A., McManigal B., Melese A., Misgina K.H., Mohamed N.S., Mohammed M., Mohammed S., Mohammed S., Mokdad A.H., Moore C.E., Mougin V., Mturi N., Mulugeta T., Musaigwa F., Musicha P., Musila L.A., Muthupandian S., Naghavi P., Negash H., Nuckchady D.C., Obiero C.W., Odetokun I.A., Ogundijo O.A., Okidi L., Okonji O.C., Olagunju A.T., Olufadewa I.I., Pak G.D., Perovic O., Pollard A., Raad M., Rafaï C., Ramadan H., Redwan E.M.M., Roca A., Rosenthal V.D., Saleh M.A., Samy A.M., Sharland M., Shittu A., Siddig E.E., Sisay E.A., Stergachis A., Tesfamariam W.B., Tigoi C., Tincho M.B., Tiruye T.Y., Umeokonkwo C.D., Walsh T., Walson J.L., Yusuf H., Zeru N.G., Hay S.I., Dolecek C., Murray C.J.L., Naghavi M. (2024). The burden of bacterial antimicrobial resistance in the WHO African region in 2019: a cross-country systematic analysis. Lancet Glob. Health.

[3] Naghavi M., Vollset S.E., Ikuta K.S., Swetschinski L.R., Gray A.P., Wool E.E., Aguilar G. Robles, Mestrovic T., Smith G., Han C., Hsu R.L., Chalek J., Araki D.T., Chung E., Raggi C., Hayoon A. Gershberg, Weaver N. Davis, Lindstedt P.A., Smith A.E., Altay U., Bhattacharjee N.V., Giannakis K., Fell F., McManigal B., Ekapirat N., Mendes J.A., Runghien T., Srimokla O., Abdelkader A., Abd-Elsalam S., Aboagye R.G., Abolhassani H., Abualruz H., Abubakar U., Abukhadijah H.J., Aburuz S., Abu-Zaid A., Achalapong S., Addo I.Y., Adekanmbi V., Adeyeoluwa T.E., Adnani Q.E.S., Adzigbli L.A., Afzal M.S., Afzal S., Agodi A., Ahlstrom A.J., Ahmad A., Ahmad S., Ahmad T., Ahmadi A., Ahmed A., Ahmed H., Ahmed I., Ahmed M., Ahmed S., Ahmed S.A., Akkaif M.A., Al Awaidy S., Al Thaher Y., Alalalmeh S.O., AlBataineh M.T., Aldhaleei W.A., Al-Gheethi A.A.S., Alhaji N.B., Ali A., Ali L., Ali S.S., Ali W., Allel K., Al-Marwani S., Alrawashdeh A., Altaf A., Al-Tammemi A.B., Al-Tawfiq J.A., Alzoubi K.H., Al-Zyoud W.A., Amos B., Amuasi J.H., Ancuceanu R., Andrews J.R., Anil A., Anuoluwa I.A., Anvari S., Anyasodor A.E., Apostol G.L.C., Arabloo J., Arafat M., Aravkin A.Y., Areda D., Aremu A., Artamonov A.A., Ashley E.A., Asika M.O., Athari S.S., Atout M.M.W., Awoke T., Azadnajafabad S., Azam J.M., Aziz S., Azzam A.Y., Babaei M., Babin F.-X., Badar M., Baig A.A., Bajcetic M., Baker S., Bardhan M., Barqawi H.J., Basharat Z., Basiru A., Bastard M., Basu S., Bayleyegn N.S., Belete M.A., Bello O.O., Beloukas A., Berkley J.A., Bhagavathula A.S., Bhaskar S., Bhuyan S.S., Bielicki J.A., Briko N.I., Brown C.S., Browne A.J., Buonsenso D., Bustanji Y., Carvalheiro C.G., Castañeda-Orjuela C.A., Cenderadewi M., Chadwick J., Chakraborty S., Chandika R.M., Chandy S., Chansamouth V., Chattu V.K., Chaudhary A.A., Ching P.R., Chopra H., Chowdhury F.R., Chu D.-T., Chutiyami M., Cruz-Martins N., da Silva A.G., Dadras O., Dai X., Darcho S.D., Das S., De la Hoz F.P., Dekker D.M., Dhama K., Diaz D., Dickson B.F.R., Djorie S.G., Dodangeh M., Dohare S., Dokova K.G., Doshi O.P., Dowou R.K., Dsouza H.L., Dunachie S.J., Dziedzic A.M., Eckmanns T., Ed-Dra A., Eftekharimehrabad A., Ekundayo T.C., El Sayed I., Elhadi M., El-Huneidi W., Elias C., Ellis S.J., Elsheikh R., Elsohaby I., Eltaha C., Eshrati B., Eslami M., Eyre D.W., Fadaka A.O., Fagbamigbe A.F., Fahim A., Fakhri-Demeshghieh A., Fasina F.O., Fasina M.M., Fatehizadeh A., Feasey N.A., Feizkhah A., Fekadu G., Fischer F., Fitriana I., Forrest K.M., Rodrigues C. Fortuna, Fuller J.E., Gadanya M.A., Gajdács M., Gandhi A.P., Garcia-Gallo E.E., Garrett D.O., Gautam R.K., Gebregergis M.W., Gebrehiwot M., Gebremeskel T.G., Geffers C., Georgalis L., Ghazy R.M., Golechha M., Golinelli D., Gordon M., Gulati S., Gupta R. Das, Gupta S., Gupta V.K., Habteyohannes A.D., Haller S., Harapan H., Harrison M.L., Hasaballah A.I., Hasan I., Hasan R.S., Hasani H., Haselbeck A.H., Hasnain M.S., Hassan I.I., Hassan S., Tabatabaei M.S. Hassan Zadeh, Hayat K., He J., Hegazi O.E., Heidari M., Hezam K., Holla R., Holm M., Hopkins H., Hossain M.M., Hosseinzadeh M., Hostiuc S., Hussein N.R., Huy L.D., Ibáñez-Prada E.D., Ikiroma A., Ilic I.M., Islam S.M.S., Ismail F., Ismail N.E., Iwu C.D., Iwu-Jaja C.J., Jafarzadeh A., Jaiteh F., Jalilzadeh Yengejeh R., Jamora R.D.G., Javidnia J., Jawaid T., Jenney A.W.J., Jeon H.J., Jokar M., Jomehzadeh N., Joo T., Joseph N., Kamal Z., Kanmodi K.K., Kantar R.S., Kapisi J.A., Karaye I.M., Khader Y.S., Khajuria H., Khalid N., Khamesipour F., Khan A., Khan M.J., Khan M.T., Khanal V., Khidri F.F., Khubchandani J., Khusuwan S., Kim M.S., Kisa A., Korshunov V.A., Krapp F., Krumkamp R., Kuddus M., Kulimbet M., Kumar D., Kumaran E.A.P., Kuttikkattu A., Kyu H.H., Landires I., Lawal B.K., Le T.T.T., Lederer I.M., Lee M., Lee S.W., Lepape A., Lerango T.L., Ligade V.S., Lim C., Lim S.S., Limenh L.W., Liu C., Liu X., Liu X., Loftus M.J., Amin H.I.M., Maass K.L., Maharaj S.B., Mahmoud M.A., Maikanti-Charalampous P., Makram O.M., Malhotra K., Malik A.A., Mandilara G.D., Marks F., Martinez-Guerra B.A., Martorell M., Masoumi-Asl H., Mathioudakis A.G., May J., McHugh T.A., Meiring J., Meles H.N., Melese A., Melese E.B., Minervini G., Mohamed N.S., Mohammed S., Mohan S., Mokdad A.H., Monasta L., Ghalibaf A. Moodi, Moore C.E., Moradi Y., Mossialos E., Mougin V., Mukoro G.D., Mulita F., Muller-Pebody B., Murillo-Zamora E., Musa S., Musicha P., Musila L.A., Muthupandian S., Nagarajan A.J., Naghavi P., Nainu F., Nair T.S., Najmuldeen H.H.R., Natto Z.S., Nauman J., Nayak B.P., Nchanji G.T., Ndishimye P., Negoi I., Negoi R.I., Nejadghaderi S.A., Nguyen Q.P., Noman E.A., Nwakanma D.C., O’Brien S., Ochoa T.J., Odetokun I.A., Ogundijo O.A., Ojo-Akosile T.R., Okeke S.R., Okonji O.C., Olagunju A.T., Olivas-Martinez A., Olorukooba A.A., Olwoch P., Onyedibe K.I., Ortiz-Brizuela E., Osuolale O., Ounchanum P., Oyeyemi O.T., A M.P.P., Paredes J.L., Parikh R.R., Patel J., Patil S., Pawar S., Peleg A.Y., Peprah P., Perdigão J., Perrone C., Petcu I.-R., Phommasone K., Piracha Z.Z., Poddighe D., Pollard A.J., Poluru R., Ponce-De-Leon A., Puvvula J., Qamar F.N., Qasim N.H., Rafai C.D., Raghav P., Rahbarnia L., Rahim F., Rahimi-Movaghar V., Rahman M., Rahman M.A., Ramadan H., Ramasamy S.K., Ramesh P.S., Ramteke P.W., Rana R.K., Rani U., Rashidi M.-M., Rathish D., Rattanavong S., Rawaf S., Redwan E.M.M., Reyes L.F., Roberts T., Robotham J.V., Rosenthal V.D., Ross A.G., Roy N., Rudd K.E., Sabet C.J., Saddik B.A., Saeb M.R., Saeed U., Saeedi Moghaddam S., Saengchan W., Safaei M., Saghazadeh A., Sharif-Askari N. Saheb, Sahebkar A., Sahoo S.S., Sahu M., Saki M., Salam N., Saleem Z., Saleh M.A., Samodra Y.L., Samy A.M., Saravanan A., Satpathy M., Schumacher A.E., Sedighi M., Seekaew S., Shafie M., Shah P.A., Shahid S., Shahwan M.J., Shakoor S., Shalev N., Shamim M.A., Shamshirgaran M.A., Shamsi A., Sharifan A., Shastry R.P., Shetty M., Shittu A., Shrestha S., Siddig E.E., Sideroglou T., Sifuentes-Osornio J., Silva L.M.L.R., Simões E.A.F., Simpson A.J.H., Singh A., Singh S., Sinto R., Soliman S.S.M., Soraneh S., Stoesser N., Stoeva T.Z., Swain C.K., Szarpak L., Y S.S.T., Tabatabai S., Tabche C., Taha Z.M.-A., Tan K.-K., Tasak N., Tat N.Y., Thaiprakong A., Thangaraju P., Tigoi C.C., Tiwari K., Tovani-Palone M.R., Tran T.H., Tumurkhuu M., Turner P., Udoakang A.J., Udoh A., Ullah N., Ullah S., Vaithinathan A.G., Valenti M., Vos T., Vu H.T.L., Waheed Y., Walker A.S., Walson J.L., Wangrangsimakul T., Weerakoon K.G., Wertheim H.F.L., Williams P.C.M., Wolde A.A., Wozniak T.M., Wu F., Wu Z., Yadav M.K.K., Yaghoubi S., Yahaya Z.S., Yarahmadi A., Yezli S., Yismaw Y.E., Yon D.K., Yuan C.-W., Yusuf H., Zakham F., Zamagni G., Zhang H., Zhang Z.-J., Zielińska M., Zumla A., Zyoud S.H.H., Zyoud S.H., Hay S.I., Stergachis A., Sartorius B., Cooper B.S., Dolecek C., Murray C.J.L. (2024). Global burden of bacterial antimicrobial resistance 1990–2021: a systematic analysis with forecasts to 2050. Lancet.

[4] Croxen M.A., Finlay B.B. (2010). Molecular mechanisms of *Escherichia coli* pathogenicity. Nat. Rev. Microbiol..

[5] Kaper J.B., Nataro J.P., Mobley H.L.T. (2004). Pathogenic *Escherichia coli*. Nat. Rev. Microbiol..

[6] Olaitan M.O., Orababa O.Q., Shittu R.B., Obunukwu G.M., Kade A.E., Arowolo M.T., Oyediran A.A., Yusuff R.A. (2025). Prevalence of ESBL-producing *Escherichia coli* in sub-Saharan Africa: a meta-analysis using a one health approach. One Heal..

[7] Lester R., Musicha P., Kawaza K., Langton J., Mango J., Mangochi H., Bakali W., Pearse O., Mallewa J., Denis B., Bilima S., Gordon S.B., Lalloo D.G., Jewell C.P., Feasey N.A. (2022). Effect of resistance to third-generation cephalosporins on morbidity and mortality from bloodstream infections in Blantyre, Malawi: a prospective cohort study. Lancet Microbe.

[8] Ndir A., Diop A., Ka R., Faye P.M., Dia-Badiane N.M., Ndoye B., Astagneau P. (2016). Infections caused by extended-spectrum beta-lactamases producing Enterobacteriaceae: clinical and economic impact in patients hospitalized in 2 teaching hospitals in Dakar, Senegal. Antimicrob. Resist. Infect. Control.

[9] World Health Organization (2024). WHO Bacterial Priority Pathogens List, 2024: Bacterial Pathogens of Public Health Importance to Guide Research, Development and Strategies to Prevent and Control Antimicrobial Resistance, Geneva, Switzerland. https://www.who.int/publications/i/item/9789240093461.

[10] Kocsis B., Gulyás D., Szabó D. (2022). Emergence and dissemination of extraintestinal pathogenic high-risk international clones of *Escherichia coli*. Life.

[11] Petty N.K., Ben Zakour N.L., Stanton-Cook M., Skippington E., Totsika M., Forde B.M., Phan M.-D., Gomes Moriel D., Peters K.M., Davies M., Rogers B.A., Dougan G., Rodriguez-Baño J., Pascual A., Pitout J.D.D., Upton M., Paterson D.L., Walsh T.R., Schembri M.A., Beatson S.A. (2014). Global dissemination of a multidrug resistant *Escherichia coli* clone. Proc. Natl. Acad. Sci. USA.

[12] Ikhimiukor O.O., Odih E.E., Donado-Godoy P., Okeke I.N. (2022). A bottom-up view of antimicrobial resistance transmission in developing countries. Nat. Microbiol..

[13] Manenzhe R.I., Zar H.J., Nicol M.P., Kaba M. (2015). The spread of carbapenemase-producing bacteria in Africa: a systematic review. J. Antimicrob. Chemother..

[14] Eger E., Heiden S.E., Korolew K., Bayingana C., Ndoli J.M., Sendegeya A., Gahutu J.B., Kurz M.S.E., Mockenhaupt F.P., Müller J., Simm S., Schaufler K. (2021). Circulation of extended-spectrum beta-lactamase-producing *Escherichia coli* of pandemic sequence types 131, 648, and 410 among hospitalized patients, caregivers, and the community in Rwanda. Front. Microbiol..

[15] Gay N., Rabenandrasana M.A.N., Panandiniaina H.P., Rakotoninidrina M.F., Ramahatafandry I.T., Enouf V., Roger F., Collard J.-M., Cardinale E., Rieux A., Loire E. (2023). One Health compartment analysis of ESBL-producing Escherichia coli reveals multiple transmission events in a rural area of Madagascar. J. Antimicrob. Chemother..

[16] Tricco A.C., Lillie E., Zarin W., O’Brien K.K., Colquhoun H., Levac D., Moher D., Peters M.D.J., Horsley T., Weeks L., Hempel S., Akl E.A., Chang C., McGowan J., Stewart L., Hartling L., Aldcroft A., Wilson M.G., Garritty C., Lewin S., Godfrey C.M., Macdonald M.T., Langlois E.V., Soares-Weiser K., Moriarty J., Clifford T., Tunçalp Ö., Straus S.E. (2018). PRISMA extension for scoping reviews (PRISMA-ScR): checklist and explanation. Ann. Intern. Med..

[17] African Union (2026). Member States Country Profiles. https://au.int/en/member_states/countryprofiles2.

[18] Veritas Health Innovation (2026). Covidence Systematic Review Software. https://www.covidence.org.

[19] Mohamed H.S., Galal L., Hayer J., Benavides J.A., Bañuls A.-L., Dupont C., Conquet G., Carrière C., Dumont Y., Didelot M.-N., Michon A.-L., Jean-Pierre H., Aboubaker M.H., Godreuil S. (2023). Genomic epidemiology of carbapenemase-producing gram-negative bacteria at the human-animal-environment interface in Djibouti city, Djibouti. Sci. Total Environ..

[20] Musicha P., Beale M.A., Cocker D., Oruru F.A., Zuza A., Salifu C., Katende G., Nanono S., Isaasi F., Chidziwisano K., Mugisha L., Kajumbula H., Musoke D., Morse T., Jacob S.T., Feasey N.A., Thomson N.R. (2026). Transmission of extended spectrum β-lactamase-producing Escherichia coli and antimicrobial resistance gene flow across One Health compartments in eastern Africa: a whole-genome sequence analysis from a prospective cohort study. Lancet Microbe.

[21] Milenkov M., Proux C., Rasolofoarison T.L., Rakotomalala F.A., Rasoanandrasana S., Rahajamanana V.L., Rafalimanana C., Ravaoarisaina Z., Ramahatafandry I.T., Westeel E., Petitjean M., Berti V., Marin J., Mullaert J., Han L., Clermont O., Raskine L., Endtz H., Andremont A., Denamur E., Komurian-Pradel F., Samison L.H., Armand-Lefevre L. (2024). Implementation of the WHO tricycle protocol for surveillance of extended-spectrum β-lactamase producing Escherichia coli in humans, chickens, and the environment in Madagascar: a prospective genomic epidemiology study. Lancet Microbe.

[22] Geuther N., Mbarushimana D., Habarugira F., JD B., Kollatzsch M., Pfüller R., Mugabowindekwe M., Ndoli J., FP M. (2023). ESBL-producing Enterobacteriaceae in a rural Rwandan community: carriage among community members, livestock, farm products and environment. Trop. Med. Int. Health.

[23] Muleme J., Kankya C., Munyeme M., Musoke D., Ssempebwa J.C., Isunju J.B., Wambi R., Balugaba B.E., Sekulima T., Mugambe R.K., Cadmus S., Kajumbula H.M. (2023). Phenotypic characterization and antibiograms of extended-spectrum beta-lactamase-producing Escherichia coli isolated at the human-animal-environment Interface using a one health approach among households in Wakiso District, Uganda. Infect. Drug Resist..

[24] Kavulavu B.M., Omwenga E.O., Nyangiri O.A., Nyerere A.K., Too R.J., Matey E.J., Göpel S., Mogoa W., Schilling T., Hoelzle L.E., Gladstone B.P. (2025). One health approach unravels worrying antimicrobial resistance patterns: a cross-sectional study in Kisii, Kenya. PLoS One.

[25] Iramiot J.S., Kajumbula H., Bazira J., De Villiers E.P., Asiimwe B.B. (2020). Whole genome sequences of multi-drug resistant Escherichia coli isolated in a Pastoralist Community of Western Uganda: phylogenomic changes, virulence and resistant genes. PLoS One.

[26] Mathers A.J., Peirano G., Pitout J.D.D. (2015). Adv. Appl. Microbiol..

[27] Gebru G.G., Muthupandian S., Kassaye E. (2025). Prevalence and antimicrobial resistance of *Escherichia coli* and *Klebsiella* spp in dairy farm in the Tigray region, Northern Ethiopia. BMC Microbiol..

[28] N A. Catherine, Claudia S., Savino A., Edgar M.M., Rogers K., Julius L.B., Morgan A., Imelda T.K., Joel B., Frederick B., Andreas T. (2024). Antibiotic resistance of *E. coli* isolates from different water sources in Mbarara, Uganda. J. Water Health.

[29] Chilanga F., Kasozi K.I., Mazeri S., Paterson G.K., Muwonge A. (2025). A systematic review of antimicrobial resistance transmission inferences at the human-livestock interface in Africa. Npj Antimicrob. Resist..

[30] Somda N.S., Nyarkoh R., Kotey F.C.N., Tetteh-Quarcoo P.B., Donkor E.S. (2024). A systematic review and meta-analysis of carbapenem-resistant Enterobacteriaceae in West Africa. BMC Med. Genet..

[31] Yartey S.N.-A., Kungu F., Asantewaa A.A., Donkor E.S. (2025). Extended spectrum beta-lactamase-producing bacterial clones in West Africa: a systematic review and meta-analysis from a one health perspective. Sci. Rep..

[32] Byarugaba D.K., Erima B., Wokorach G., Alafi S., Kibuuka H., Mworozi E., Musinguzi A.K., Kiyengo J., Najjuka F., Wabwire-Mangen F. (2023). Resistome and virulome of high-risk pandemic clones of multidrug-resistant extra-intestinal pathogenic Escherichia coli (ExPEC) isolated from tertiary healthcare settings in Uganda. PLoS One.

[33] Nnah E.P., Asante J., Amoako D.G., Abia A.L.K., Essack S.Y. (2025). Antibiotic-resistant *Escherichia coli* (E. coli) at one health interfaces in Africa: a scoping review. Sci. Total Environ..

[34] Karanika S., Karantanos T., Arvanitis M., Grigoras C., Mylonakis E. (2016). Fecal colonization with extended-spectrum beta-lactamase–producing Enterobacteriaceae and risk factors among healthy individuals: a systematic review and metaanalysis. Clin. Infect. Dis..

[35] Storberg V. (2014). ESBL-producing Enterobacteriaceae in Africa – a non-systematic literature review of research published 2008–2012. Infect. Ecol. Epidemiol..

