# Supplementary material for: Extended spectrum beta lactamases and carbapenemase-producing Escherichia coli in humans, animals and the environment in East Africa: a scoping review
Source: One Health. 2026 Oct 10;23:101606. doi: 10.1016/j.onehlt.2026.101606 (PMC13683449; doi:10.1016/j.onehlt.2026.101606)
Supplement: Supplementary file 1 [file mmc1.docx]

**SUPPLEMENTARY MATERIALS**

**Table S1.** Electronic database and search strings used to identify studies on ESBL and carbapenemase-producing *E. coli* in East Africa (01/2015-12/2025) for the scoping review

| **Database** | **Search string** |
| --- | --- |
| PubMed (n =164) | (((("*Escherichiacoli*"[Title/Abstract] OR UPEC[Title/Abstract] OR uropathogenic[Title/Abstract] OR ExPEC[Title/Abstract] OR "extraintestinal pathogenic *Escherichia coli*"[Title/Abstract] OR "extraintestinal *E. coli*"[Title/Abstract] ) NOT ( EPEC[Title/Abstract] OR ETEC[Title/Abstract] OR EAEC[Title/Abstract] OR EHEC[Title/Abstract] OR STEC[Title/Abstract] OR EIEC[Title/Abstract] OR EAggEC[Title/Abstract] OR diarrheagenic[Title/Abstract] ) ) AND (ESBL[Title/Abstract] OR "extended-spectrum beta-lactamase"[Title/Abstract] OR carbapenemase*[Title/Abstract] ) AND ( UTI[Title/Abstract] OR urine[Title/Abstract] OR urinary[Title/Abstract] OR bloodstream[Title/Abstract] OR sepsis[Title/Abstract] OR extraintestinal[Title/Abstract] OR infection[Title/Abstract] OR human*[Title/Abstract] OR animal*[Title/Abstract] OR environment*[Title/Abstract] OR wastewater[Title/Abstract] OR water[Title/Abstract] OR livestock[Title/Abstract] OR poultry[Title/Abstract] OR fish[Title/Abstract] OR "One Health"[Title/Abstract] ) AND (Tanzania[Title/Abstract] OR Kenya[Title/Abstract] OR Uganda[Title/Abstract] OR Rwanda[Title/Abstract] OR Burundi[Title/Abstract] OR Ethiopia[Title/Abstract] OR Eritrea[Title/Abstract] OR Somalia[Title/Abstract] OR Djibouti[Title/Abstract] OR Sudan[Title/Abstract] OR "South Sudan"[Title/Abstract] OR Comoros[Title/Abstract] OR Seychelles[Title/Abstract] OR Mauritius[Title/Abstract] OR Madagascar[Title/Abstract] OR Malawi[Title/Abstract] OR Mozambique[Title/Abstract] ) ) AND ("2015/01/01"[Date - Publication] : "2025/12/31"[Date - Publication])) NOT (Review[Publication Type] OR Editorial[Publication Type] OR Letter[Publication Type] OR Comment[Publication Type] OR Meta-Analysis[Publication Type] ) |
| Scopus (n =115) | TITLE-ABS-KEY ( ( ( ExPEC OR "extraintestinal pathogenic *Escherichia coli*" OR UPEC OR uropathogenic OR "extraintestinal" ) OR ( ( "*Escherichia coli*" ) AND ( UTI OR urinary OR urine OR bloodstream OR sepsis OR invasive ) ) ) AND NOT ( EPEC OR ETEC OR EAEC OR EHEC OR STEC OR EIEC OR EAggEC OR DEC OR diarrheagenic ) AND ( ESBL OR "extended-spectrum beta-lactamase" OR carbapenemase* ) AND ( human* OR animal* OR environment* OR wastewater OR water OR livestock OR poultry OR fish OR "One Health" ) AND ( Tanzania OR Kenya OR Uganda OR Rwanda OR Burundi OR Ethiopia OR Eritrea OR Somalia OR Djibouti OR Sudan OR "South Sudan" OR Comoros OR Seychelles OR Mauritius OR Madagascar) ) OR Malawi OR Mozambique ) ) AND PUBYEAR > 2014 AND PUBYEAR < 2026 AND NOT DOCTYPE ( re ) AND NOT DOCTYPE ( ed ) AND NOT DOCTYPE ( cp ) AND NOT DOCTYPE ( no ) AND NOT SRCTYPE ( k ) |

**Table S2.** Data variables and extraction parameters

| **Variable** | **Extracted data** |
| --- | --- |
| Study identification | First author; year of publication; title of study; country of study |
| Study design | Study design (e.g., cross-sectional, cohort, surveillance) |
| Study setting | Hospital, community, animal, and/or environmental setting |
| Population and sampling | Study population; sample size; study period (years of data collection) |
| Sample information | Sample type (e.g., urine, stool, blood, food, water, soil etc.) |
| Bacterial characteristics | Bacterial species identified; number of bacterial isolates |
| Bacterial identification methods | Identification methods (e.g., culture, MALDI-TOF, VITEK2) |
| Diagnostic methods | Phenotypic and/or molecular diagnostic methods |
| Antimicrobial Susceptibility Testing (AST) | AST methods (disk diffusion, MIC, automated systems); Guidelines used (CLSI or EUCAST) |
| Molecular Analysis | Molecular methods (PCR, WGS, etc.); ESBL and carbapenemase genes (e.g., CTX-M, TEM, SHV, NDM, KPC, OXA-48, VIM, IMP); phylogroups, plasmid replicons, sequence types |
| ESBLs | ESBL reported (yes/no); ESBL prevalence (%) |
| Carbapenemase | Carbapenemase reported (yes/no); Carbapenem resistance prevalence (%) |
| Antimicrobial resistance profiles | Resistance patterns and multidrug resistance profiles |
| Epidemiological factors | Risk factors associated with resistance emergence and spread |
| Key findings | Main findings (summary of results); notable patterns (e.g., outbreaks, high prevalence, cross-sector spread) |
| Study quality | Study limitations reported |

**Table S3**. Characteristics of One Health studies (n = 19) across human, animal, food and environmental sectors

| **Study** | **Country** | **Sample sector(s)** | **Setting** | **Study design** |
| --- | --- | --- | --- | --- |
| Eger 2021 [14] | Rwanda | Human, Animal, Environment | Hospital(inpatient), community, livestock/pets of patient-caregiver households | Cross-sectional |
| Iramiot 2020 [25] | Uganda | Human, Animal | Community/household, cattle (pastoralist farms) | Cross-sectional |
| Gebru 2025 [28] | Ethiopia | Human, Animal, Food, Environment | Dairy farm, food processing facility, drinking water source, community | Cross-sectional |
| Muleme 2023 [23] (Wakiso) | Uganda | Human, Animal, Environment | Household (mixed-species livestock), household, soil/water/equipment | Cross-sectional |
| Mwakyoma 2023 [37] | Tanzania | Human, Animal, Environment | Community, farm, household/community environment (bathrooms, waste sites) | Cross-sectional |
| Sonola 2022 [38] | Tanzania | Human, Animal, Environment | Community/household, rodents/chicken, household soil | Cross-sectional |
| Musicha 2025 [20] | Uganda | Human, Animal, Food, Environment | Household (urban/peri-urban/rural),livestock/companion animals, household water, river/stream | Cohort |
| Muhummed 2024 [39] | Ethiopia | Human, Animal | Community, farm (rural children and livestock) | Cross-sectional |
| Bedane 2024 [40] | Ethiopia | Human, Animal, Environment | Outpatient/primary healthcare, aquaculture/fish farm, lake water | Cross-sectional |
| Gashaw 2025 [41] | Ethiopia | Human, Animal, Environment | Hospital (in/outpatient), community, farm, hospital environment | Cross-sectional |
| Mayanja 2023 [42] | Uganda | Human, Environment | Hospital (maternity ward), hospitalenvironment/surfaces | Cross-sectional |
| Geuther 2023 [22] | Rwanda | Human, Animal, Food, Environment | Community, farm, food vendor/street food, drinking water/soil | Cross-sectional |
| Too 2024 [43] | Kenya | Animal, Environment | Slaughterhouse, wastewater treatment plant, river/stream | Surveillance |
| Kavulavu 2025 [24] | Kenya | Human, Animal, Environment | Outpatient hospital, farm, drinking water source | Cross-sectional |
| Gay 2023 [24] | Madagascar | Human, Animal, Environment | Community, farm, drinking water source | Cross-sectional |
| Sserwadda 2023 [44] | Uganda | Human, Environment | Hospital (in/outpatient), hospital environment | Cohort |
| Mohamed 2023 [19] | Djibouti | Human, Animal, Food, Environment | Hospital, community, farm, market (fish), septic tank | Surveillance |
| Milenkov 2024 [21] | Madagascar | Human, Animal, Environment | Hospital (in/outpatient), farm/slaughterhouse (chicken), river/wastewater | Cross-sectional |
| Muleme 2023 (central Uganda) [45] | Uganda | Human, Animal, Environment | Household, farm (mixed husbandry), household environment | Cross-sectional |

**Table S4**. Temporal trend in median ESBL and carbapenemase prevalence in *E. coli* by publication period

| **Publication period** | **Median ESBL prevalence (%)** | **Median carbapenemase prevalence (%)** |
| --- | --- | --- |
| 2015-2017 | 38 | 1.8 |
| 2018-2020 | 39.7 | 0.7 |
| 2021-2023 | 50.4 | 3.3 |
| 2024-2025 | 42.8 | 7.5 |

Note: Study-level median prevalence across periods; not a regional temporal trend.

**Table S5**. Predominant *E. coli* sequence types identified across MLST-tested studies (n=31)

| **Sequence type** | **Number of studies, n** | **Percentage (%) of MLST-tested studies** |
| --- | --- | --- |
| ST131 | 17 | 54.8 |
| ST10 | 13 | 41.9 |
| ST38 | 13 | 41.9 |
| ST410 | 13 | 41.9 |
| ST167 | 10 | 32.3 |
| ST617 | 8 | 25.8 |
| ST48 | 7 | 22.6 |
| ST648 | 7 | 22.6 |
| ST69 | 5 | 16.1 |
| ST58 | 5 | 16.1 |
| ST405 | 5 | 16.1 |

**Table S6**. **Distribution of *Escherichia coli* sequence types (STs) across One Health sector combinations.**

| **Sector combination** | **Sequence types (ST)** |
| --- | --- |
| Human | ST44, ST361, ST8881, ST130, ST2172, ST219, ST295, ST345, ST517, ST641, ST683, ST2161, ST2689, ST1303, ST1585, ST393, ST906, ST2064, ST2509, ST95, ST297, ST394, ST2083, ST2178, ST2, ST43, ST53, ST244, ST500, ST690, ST692, ST698, ST741, ST829, ST954, ST960 |
| Human + Animal | ST224, ST5614, ST215, ST315, ST5204, ST2353, ST4750 |
| Human + Environment | ST1193, ST3580, ST448, ST542, ST656, ST695, ST998, ST6438 |
| Animal | ST6636, ST7460, ST58, ST196, ST349, ST1788, ST2614, ST3076, ST5909, ST9849, ST14130, ST14131, ST7588 |
| Environment | ST9549 |
| Human + Animal + Environment | ST131, ST10, ST38, ST410, ST167, ST617, ST648, ST58, ST405, ST69, ST636, ST450, ST206, ST46, ST1284, ST2852, ST2450, ST3489, ST2659, ST2851, ST156, ST226, ST457, ST540, ST155, ST716, ST1312, ST1282, ST354, ST382, ST744, ST1421, ST73, ST113, ST216, ST409, ST452, ST773, ST10447, ST2690, ST2705, ST940, ST1431, ST2914, ST13464, ST602, ST12780 |
| Animal + Environment | None reported |

**Table S7.** Distribution of *E. coli* phylogroups across phylotyped studies (n=22)

| **Phylogroup** | **Number of studies** | **Percentage (%) of phylotyped studies** |
| --- | --- | --- |
| A | 19 | 86.4 |
| B1 | 17 | 77.3 |
| B2 | 13 | 59.1 |
| D | 13 | 59.1 |
| C | 12 | 54.5 |
| F | 12 | 54.5 |
| E | 7 | 31.8 |
| G | 3 | 13.6 |
| Clade I | 3 | 13.6 |

**Table S8.** Predominant plasmid replicon types identified across replicon-typed studies (n=21)

| **Plasmid replicon** | **Number of studies** | **Percentage (%) of replicon-tested studies** |
| --- | --- | --- |
| IncFIA | 12 | 57.1 |
| IncFII | 12 | 57.1 |
| IncFIB | 8 | 38.1 |
| IncY | 8 | 38.1 |
| Col (BS512) | 6 | 28.6 |
| IncI1 | 6 | 28.6 |
| IncQ1 | 6 | 28.6 |
| Col156 | 5 | 23.8 |
| IncB/O/K/Z | 5 | 23.8 |
| IncFIB (AP001918) | 5 | 23.8 |

**Table S9. Predominant virulence genes identified across virulence-tested studies (n=25)**

| **Virulence gene** | **Functional category** | **Number of studies** | **Percentage (%) of virulence-tested studies** |
| --- | --- | --- | --- |
| *iss* | Serum resistance | 9 | 36.0 |
| *gad* | Acid/stress resistance | 8 | 32.0 |
| *lpfA* | Adhesion (fimbriae) | 8 | 32.0 |
| *traT* | Serum/complement resistance | 8 | 32.0 |
| *iutA* | Iron acquisition (aerobactin) | 7 | 28.0 |
| *air* | Adhesion | 6 | 24.0 |
| *capU* | Cell-surface associated | 6 | 24.0 |
| *eilA* | Invasion regulator | 6 | 24.0 |
| *fimH* | Adhesion (type 1 fimbriae) | 6 | 24.0 |
| *fyuA* | Iron acquisition (yersiniabactin) | 6 | 24.0 |
| *ompT* | Protease/serum resistance | 6 | 24.0 |
| *sat* | Toxin (autotransporter) | 6 | 24.0 |
| *terC* | Stress/metal resistance | 6 | 24.0 |

**Table S10.** Risk factors investigated and identified, organized by category and sector

| **Category** | **Studies** | **Key variables/findings** |
| --- | --- | --- |
| Antibiotic use (human) | Sahle 2022 [46]; Kurz 2017 [47]; Amsalu 2024 [48] ; Tadesse 2022 [49]; Diriba 2020 [50]; Belete 2020 [51] | Prior antibiotic use (1-6 months) consistently significant for ESBL/Carbapenemase carriage |
| Healthcare exposure (human) | Alebel 2021 [52]; Asmare 2024 [53] ; Manyahi 2022 [54]; Kiponza 2019 [55]; Mohamed 2022 [56] | Hospitalization, catheterization, ICU/NICU admission, invasive devices, prolonged length of stay |
| Age / host factors (human) | Tellevik 2016 [57]; Kibwana 2022 [58]; Sewunet 2022 [59]; Fenta 2020 [60] | Young age (<5 years, especially <12 months); male uncircumcision (paediatric UTI) |
| Sex/demographic (human) | Gebremariam 2019 [61] ; Tumuhamye 2021 [62]; Bazira 2025 [63] ; El-Arifi 2024 [64] | Female sex, prior UTI history, frequent sexual activity, maternal age ≥30 |
| Comorbidities/immune status (human) | Simeneh 2022 [65]; Befikadu 2024 [66]; Alemu 2020 [67] | Low CD4 count, chronic disease, diabetes/hyperglycaemia, HIV-related immunosuppression |
| Animal contact / One Health (human-animal) | Kavulavu 2025 [24]; Eger 2021 [14]; Muleme 2023 [23]; Sonola 2022 [38]; Muhummed 2024 [39] | Direct animal contact, livestock/poultry keeping, houseflies as vectors |
| Water/sanitation exposure (human + environment) | Kavulavu 2025 [24]; Tola 2021 [68]; Catherine N 2024 [29]; Muhummed 2024 [39] | Unprotected/untreated drinking water, well proximity to pit latrines |
| Food-chain/food-handling exposure | Diriba 2020 [50]; Abayneh 2019 [69] ; Genet 2025 [70]; Gebru 2025 [28] | Unhygienic meat/milk handling, raw milk or meat consumption, absence of health certification |
| Animal-level factors | Ball 2019 [71]; Manishimwe 2021 [72]; Bager 2022 [73]; Akunda 2023 [74] | Animal species (poultry highest MDR), close human contact (sanctuary vs. wild wildlife) significant; production system (pastoralism vs. ranching) not significant |
| Environmental/geographic factors | Maina 2021 [75] Kimera 2021[76] | Proximity to open sewage/refuse sites; upstream-to-downstream contamination gradient |
| No significant risk factors identified | Geuther 2023 [22]; Kateregga 2015 [77]; Zain 2022 [78]; Mutuma 2023 [79]; Ndihokubwayo 2025 [80]; Felician 2025 [81] | No statistically significant predictors despite formal testing |

**Table S11**. Antimicrobial resistance phenotype of *E. coli* isolates by antibiotic (n=25)

| **Antibiotic class** | **Antibiotic** | **Studies, n** | **Isolates tested, n** | **Isolates resistant, n** | **Median resistance (%)** | **Range (%)** |
| --- | --- | --- | --- | --- | --- | --- |
| Penicillins | Amoxicillin | 8 | 2,480 | 2,214 | 97.5 | 80.0 - 100.0 |
| Penicillins | Ampicillin | 75 | 11,061 | 7,566 | 90.0 | 0.3 - 100.0 |
| β-lactam/β-lactamase inhibitor | Amoxicillin-clavulanate | 64 | 6,267 | 2,859 | 62.2 | 0.0 - 100.0 |
| β-lactam/β-lactamase inhibitor | Piperacillin-tazobactam | 31 | 2,876 | 1,057 | 22.7 | 0.0 - 100.0 |
| Cephalosporins (2^nd^ gen) | Cefuroxime | 27 | 2,598 | 1,676 | 69.5 | 1.2 - 100.0 |
| Cephalosporins (3^rd^ gen) | Cefotaxime | 60 | 7,760 | 4,078 | 52.9 | 0.0 - 100.0 |
| Cephalosporins (3^rd^ gen) | Ceftazidime | 68 | 5,912 | 2,534 | 47.9 | 0.0 - 100.0 |
| Cephalosporins (3^rd^ gen) | Ceftriaxone | 60 | 7,345 | 3,366 | 46.8 | 0.0 - 100.0 |
| Cephalosporins (4^th^ gen) | Cefepime | 39 | 3,572 | 1,447 | 44.5 | 0.0 - 100.0 |
| Monobactams | Aztreonam | 16 | 1,628 | 842 | 85.3 | 0.0 - 100.0 |
| Carbapenems | Ertapenem | 15 | 1,440 | 137 | 2.9 | 0.0 - 100.0 |
| Carbapenems | Imipenem | 42 | 4,138 | 291 | 2.3 | 0.0 - 76.9 |
| Carbapenems | Meropenem | 62 | 8,650 | 546 | 4.3 | 0.0 - 100.0 |
| Aminoglycosides | Gentamicin | 84 | 9,871 | 3,128 | 31.2 | 0.0 - 100.0 |
| Aminoglycosides | Amikacin | 35 | 3,893 | 453 | 6.0 | 0.0 - 41.3 |
| Aminoglycosides | Tobramycin | 12 | 907 | 425 | 29.8 | 0.0 - 89.5 |
| Quinolones | Nalidixic acid | 15 | 4,266 | 1,650 | 44.6 | 6.5 - 94.7 |
| Fluoroquinolones | Ciprofloxacin | 94 | 11,398 | 5,044 | 37.2 | 0.0 - 100.0 |
| Fluoroquinolones | Levofloxacin | 7 | 540 | 113 | 22.7 | 5.4 - 50.0 |
| Tetracyclines | Tetracycline | 43 | 3,569 | 2,066 | 71.4 | 0.4 - 100.0 |
| Folate pathway inhibitors | Trimethoprim-sulfamethoxazole | 90 | 11,644 | 7,435 | 69.8 | 0.4 - 100.0 |
| Phenicols | Chloramphenicol | 45 | 5,558 | 1,715 | 36.4 | 0.0 - 100.0 |
| Nitrofurans | Nitrofurantoin | 37 | 2,143 | 349 | 13.9 | 0.0 - 68.1 |
| Fosfomycins | Fosfomycin | 10 | 827 | 25 | 4.7 | 0.0 - 38.5 |
| Polymyxins | Colistin | 11 | 774 | 32 | 0.0 | 0.0 - 100.0 |

**Table S12**. Number of studies contributing to each molecular/genomic analysis (n = 154)

| Analysis | Number of studies | Percentage (%) of included studies |
| --- | --- | --- |
| ESBL gene detection | 69 | 44.8 |
| Carbapenemase gene detection | 40 | 26.0 |
| Multi-locus sequence typing (MLST) | 31 | 20.1 |
| Clermont phylogrouping | 22 | 14.3 |
| Plasmid replicon typing | 21 | 13.6 |
| Virulence gene profiling | 25 | 16.2 |
| Whole-genome sequencing (WGS) | 30 | 19.5 |

**Figure S1** Country-level heatmap; cell values indicate the percentage of gene-tested studies per country reporting each gene, with darker shading representing higher frequencies.

**Figure S2** Country-level heatmap of carbapenemase gene distribution in *E. coli*; cell values indicate the percentage of gene-tested studies per country reporting each gene, with darker shading representing higher frequencies.

**Figure S3** Heatmap showing the distribution of predominant E. coli sequence types (STs) across East African countries. Cell values indicate the percentage of MLST-tested studies reporting each ST, with the number of MLST-tested studies shown beside each country (n). Darker blue shading represents higher reporting frequencies.

**Figure S4** Heatmap showing the distribution of E. coli Clermont phylogroups across East African countries. Cell values represent the **percentage of phylogroup-tested studies** reporting each phylogroup, with the number of phylogroup-tested studies indicated in parentheses beside each country.
